# Blimp-1 and c-Maf regulate *Il10 and *negatively regulate common and unique proinflammatory gene networks in IL-12 plus IL-27-driven T helper-1 cells

**DOI:** 10.12688/wellcomeopenres.19680.1

**Published:** 2023-09-18

**Authors:** Luke S. Cox, Marisol Alvarez-Martinez, Xuemei Wu, Leona Gabryšová, Raphaëlle Luisier, James Briscoe, Nicholas M. Luscombe, Anne O'Garra

**Affiliations:** 1 Immunoregulation and Infection Laboratory, The Francis Crick Institute, London, England, NW1 1AT, UK; 2 Computational Biology Laboratory, The Francis Crick Institute, London, England, NW1 1AT, UK; 3 Developmental Dynamics Laboratory, The Francis Crick Institute, London, England, NW1 1AT, UK; 4 UCL Genetics Institute, Department of Genetics, Evolution and Environment, University College London, London, England, UK; 5 National Heart and Lung Institute, Imperial College London, London, England, UK

**Keywords:** CD4+ T cells, Th1 cells, IL-10, IFN-γ, Prdm1, Maf

## Abstract

**Background:** CD4 ^+^ Th1 cells producing IFN-γ are required to eradicate intracellular pathogens, however if uncontrolled these cells can cause immunopathology. The cytokine IL-10 is produced by multiple immune cells including Th1 cells during infection and regulates the immune response to minimise collateral host damage. In this study we aimed to elucidate the transcriptional network of genes controlling the expression of *Il10* and proinflammatory cytokines, including *Ifng* in Th1 cells.

**Methods:** We applied computational analysis of gene regulation derived from temporal profiling of gene expression clusters obtained from bulk RNA sequencing (RNA-seq) of flow cytometry sorted CD4 ^+^ Th1 effector cells differentiated *in vitro* with IL-12 and IL-27 to produce *Ifng* and *Il10,* compared to control driven-CD4+ T cells. Data were integrated with analysis of active genomic regions from these Th1 cells using an assay for transposase-accessible chromatin with sequencing (ATAC)-seq, integrated with literature derived-Chromatin-immunoprecipitation (ChIP)-seq data and the RNA-seq data, to elucidate the transcriptional network of genes controlling expression of *Il10* and pro-inflammatory effector genes in Th1 cells. The co-dominant role for the transcription factors, *Prdm1* (encoding Blimp-1) and *Maf* (encoding c-Maf), in cytokine gene regulation in Th1 cells, was confirmed using T cells obtained from mice with T-cell specific deletion of these transcription factors.

**Results:** We show that the transcription factors Blimp-1 and c-Maf each have unique and common effects on cytokine gene regulation and not only co-operate to induce *Il10* gene expression in IL-12 plus IL-27 differentiated Th1 cells, but additionally directly negatively regulate key proinflammatory cytokines including *Ifng*, thus providing mechanisms for reinforcement of regulated Th1 cell responses.

**Conclusions:** These data show that Blimp-1 and c-Maf positively and negatively regulate a network of both unique and common anti-inflammatory and pro-inflammatory genes to reinforce a Th1 response that will eradicate pathogens with minimum immunopathology.

## Introduction

CD4+ T helper 1 (Th1) cells are critical in controlling infection by production of the cytokine IFN-γ, which upregulates the expression of MHC-class II molecules on antigen-presenting cells (APC) thus enhancing their capacity to present antigen to activate CD4 ^+^ T cells, and also activates macrophages to kill intracellular pathogens ^1^. However, uncontrolled Th1 responses can cause immunopathology ^2^. IL-10 is a regulatory cytokine that has been widely shown to limit immunopathology particularly during infection and intestinal responses to pathobionts ^3– 6^ and mutations in IL-10 or the IL-10R result in inflammatory bowel disease (IBD) in humans ^7, 8^. Most cells of the immune system can produce IL-10 to limit over-exuberant immune responses and pathology ^5, 6, 9^. Th1 cells have been shown to be the critical source of IL-10 to limit responses to pathogens such as *Toxoplasma gondii* ^10^ and *Leishmania major* ^11^ and thus avoid immunopathology. The cytokine IL-27, which has been reported to regulate the immune response by multiple mechanisms ^12, 13^, promotes IL-10 production by effector Th1 CD4 ^+^ T cells *in vivo* in response to the malaria parasites *Plasmodium chabaudi* ^14^ and *L. major* ^15^ infections, providing a critical mechanism for protection from severe immunopathology. However, the transcriptional mechanisms that regulate cytokine production by Th1 cells including their production of IL-10 are as yet unclear.

Several transcription factors have been shown to regulate IL-10 in T cells ^13, 16^. Both common and cell-specific transcriptional mechanisms are in place to tightly regulate the expression of *Il10* and proinflammatory gene expression in T cells to ensure a controlled immune response to pathogens and/or other insults ^5, 6, 17– 19^. Since transcription factors have multiple gene targets this raises the question as to whether known transcription factors that positively regulate *Il10* may simultaneously negatively regulate proinflammatory cytokine expression in T cells, thus driving a controlled response to control immune responses to pathogens and pathobionts to limit host damage. For example, the transcription factor c-Maf has been shown to induce *Il10* expression directly across multiple T cell subsets both *in vitro* and *in vivo* ^5, 6, 13, 17– 19^ whilst also acting as a negative regulator of *Il2* ^16^ and Th17 responses ^20^. Blimp-1, encoded by the *Prdm1* gene, has also been shown to induce IL-10 ^13, 18, 19^, although originally described as a global regulator of T cell homeostasis and differentiation *in vivo* ^21– 25^.

Recently Kuchroo *et al.* ^13^, systematically identified regulators for *Il10* and highlighted *Prdm1* and *Maf* as two central nodes of the *Il10* regulatory circuits that cooperatively promoted IL-10 production in ‘Tr1 cells’ differentiated *in vitro* with IL-27 and through gain-of-function in Th1, Th2, Th17, and Treg cells ^13^. IL-27-driven ‘Tr1 cells’ lacking both *Prdm1* and *Maf* (DKO) showed an almost complete loss of *Il10* expression. Moreover, expression of several transcription factors shown previously to be important for *Il10* expression, including Fosl2, Hif1a, Hlx, and Notch1 ^26^, was found to be abrogated in IL-27-driven ‘Tr1 cells’ from CD4 ^+^ T cells deficient in both *Prdm1* and *Maf* ^13^. Moreover, DKO ‘Tr1 cells’ showed a unique reduction in chromatin accessibility in co-inhibitory receptor gene loci such as *Ctla4, Pdcd1* (PD-1), *Tigit, Havcr2* (Tim-3), suggesting that *Prdm1* and *Maf* have complementary but indispensable roles in regulating Tr1 at the transcriptional level and reinforcing the expression of negative immune regulators. Although IL-27 regulates the effector function of IL-12 driven Th1 cells, producing high levels of IFN-γ. by inducing the production of IL-10 to limit and control Th1-mediated immunopathology *in vivo* ^12, 14, 15, 27^, the mechanisms of transcriptional regulation of *Il10* and proinflammatory cytokines in Th1 cells are unclear.

In this study we applied computational analysis of gene regulation derived from temporal profiling of gene expression clusters integrated with analysis of active genomic regions in CD4 ^+^ Th1 effector cells differentiated with IL-27 or IL-12 alone, to elucidate the transcriptional network of genes controlling expression of *Il10* and pro-inflammatory effector genes in Th1 cells. We show that the transcription factors Blimp-1 and c-Maf each have unique and common effects on cytokine gene regulation and not only co-operate to induce *Il10* gene expression in IL-12 plus IL-27 differentiated Th1 cells, but additionally directly negatively regulate key proinflammatory cytokines including *Ifng*, thus providing mechanisms for reinforcing the regulation of Th1 cell responses. Thus, Blimp-1 and c-Maf positively and negatively regulate a network of both unique and common anti-inflammatory and pro-inflammatory genes to reinforce a Th1 response that will allow eradication of pathogens with minimum immunopathology.

## Methods

### Animals

Mice were bred and maintained under specific pathogen free conditions in accordance with the Home Office UK Animals (Scientific Procedures) Act 1986. Age-matched male or female mice were used for experiments. *Maf* ^fl/fl^ mice were provided by M. Sieweke and C. Birchmeier (Max Delbrück Centre for Molecular Medicine, Germany) ^28^ and backcrossed to C57BL/6J for 10 generations and then crossed to *Cd4* ^Cre^ mice to generate *Maf* ^fl/fl^ *Cd4* ^Cre^ mice as described in 16. *Prdm1* ^fl/fl^ mice were purchased from the Jackson Laboratory (Stock Number 008100) ^29^, and further backcrossed to C57BL/6J for four generations and then crossed to *Cd4* ^Cre^ mice to generate *Prdm1* ^fl/fl^ *Cd4* ^Cre^ mice. *Prdm1* ^fl/fl^ *Maf* ^fl/fl^ *Cd4* ^Cre^ and *Prdm1* ^fl/fl^ *Maf* ^fl/fl^ control mice were generated in-house by crossing *Maf* ^fl/fl^ *Cd4* ^Cre^ with *Prdm1* ^fl/fl^ *Cd4* ^Cre^ mice. All mouse breeding was performed under strict care and husbandry ensuring no discomfort to the mice. Breeding was carried out in accordance with UK Home Office regulations, under Project License, O’Garra *P5AF488B4,* 30 Apr 18 | Amended: 10 Jan 20 | Expired: 29 Apr 23, and were approved by The Francis Crick Institute Ethical Review Panel before each submission to the UK Home Office. This study adhered to the ARRIVE guidelines ^30^.

### Naïve CD4 ^+^ T cell sorting and *in vitro* helper T cell differentiation

For each experiment, spleens were obtained after humane killing of mice by either placing the animal into a secure chamber and filling it gradually with carbon dioxide until the animal was unconscious and until death was confirmed and then followed by cervical dislocation; or by cervical dislocation of the neck, depending on how many mice needed to be humanely killed. Spleens from a total of 5–10 age-matched mice per genotype were homogenized and incubated with unconjugated rat anti-mouse antibodies against B220 (RA3.6B2, DNAX), MHC class-II (M5/114, eBioscience) and CD8 (C291.2.43, DNAX). CD4 ^+^ T cells were then negatively enriched using magnetic beads (BioMag, Qiagen). Live naïve CD4 ^+^CD62L ^+^CD44 ^lo^CD25 ^-^ T cells were then sorted to over 95% purity using the following antibodies: CD4 (RM4-5, e450), CD8 (53-6.7, FITC), CD62L (MEL-14, PE-Cy7), CD44 (IM7, PE) and CD25 (PC61.5, APC) (all from eBioscience); and propidium iodide (final concentration 2µg/ml, Sigma) on either a MoFlo XDP or Influx flow cytometer (Beckman Coulter, Inc.). Sorted naïve CD4 ^+^ T cells were then plated at 500,000 cells/well in flat-bottom 48-well plates and stimulated with plate-bound anti-CD3 (5µg/ml, 2C11, Harlan) and soluble anti-CD28 (2µg/ml, 37.51, Harlan) for up to 4 days in the presence of no polarizing cytokines for Medium control, IL-12 (rmIL-12p70, 5ng/ml, eBioscience), IL-12+IL-27 (rmIL-12p70, 5ng/ml, eBioscience; rmIL-27, 25ng/ml, R&D); or IL-27 (rmIL-27, 25ng/ml, R&D). All cells were cultured in conditioned RPMI (BE12-702F, Lonza) supplemented with 10% (v/v) heat-inactivated FCS (Gibco), 100U/ml Pen-Strep, 2mM L-glutamine, 1mM Sodium pyruvate, 10mM HEPES (all Lonza) and 0.05mM 2-Mercaptoethanol (Sigma) in a humid incubator at 37°C with 5% carbon dioxide. For each experiment three wells were differentiated per condition to give technical triplicates from the pool of naïve CD4 ^+^ T cells per genotype.

### Quantitative RT-PCR

RNA was extracted from *in vitro* differentiated T-helper cells using the QIAShredder and RNeasy Mini Kit, or RNeasy Micro kit, both with on-column DNase digestion, according to the manufacturer’s instructions (Qiagen). Eluted RNA was then reverse transcribed using a High Capacity cDNA Reverse Transcription kit (Applied Biosystems) plus RNasin (Promega) according to the manufacturer’s instructions, followed by RNaseH (Promega) treatment for 30 min at 37°C. High-Capacity cDNA Reverse Transcription kit (Applied Biosystems) was used to convert RNA into cDNA. Samples were incubated for: 10 min 25°C, 2 hr 37°C, 5 min 85°C in a thermal cycler (Vertiti Thermo Cycler, Applied Biosystems). Residual RNA was digested by RHase H incubation (final concentration 0.03U/μl, Invitrogen) for 30 min at 37°C. Reverse transcribed cDNA was then diluted to 5ng/μl using nuclease-free water (Ambion) and stored at -80°C. TaqMan™ Assay system (Applied Biosystems) was used for RT-qPCR-analysis, reaction mix/primer probes are summarised below. Reactions were carried out in 96-well plates (Applied Biosystems) on either a 7900HT or QuantStudio 3 RT-qPCR machine (Applied Biosystems). For each experiment, RT-qPCR was always performed to confirm the deletion of either *Maf, Prdm1* or both. All genes were analysed relative to the housekeeping gene hypoxanthine phosphoribosyltransferase 1 (encoded by the *Hprt*) gene. Delta Ct (ΔCt) was calculated by taking the difference between the Ct value of the gene of interest and the Ct for *Hprt* in a given sample, which was then inputted into the following equation (1.8^- ΔCt)*10^5 to give relative gene expression. For consistency, in the Applied Biosystems qPCR software the Ct threshold was manually set to 0.25 with automatic baseline threshold activated for all experiments and primer probes. cDNA was then analysed for the expression of specific genes on a 7900HT ABI, QS3 or QS5 real-time PCR system, using the TaqMan Universal Master Mix II – no UNG and the following TaqMan mouse probes (all from Applied Biosystems): *Il10* (mm00439616_m1), *Ifng* (mm01168134_m1), *Tbx21* (mm00450960_m1), *Hprt* (mm03024075_m1). All expression levels were normalised to the internal housekeeping gene *Hprt* and calculated as 1.8 ^-(Ct *Hprt*−Ct *gene*)^x10 ^5^. Reactions are run on a Thermal Cycler, Veriti model (Applied Biosystems).

### Statistical analysis

All figure legends show the number of independent biological experiments performed for each analysis and replicates. For PCR analysis, two-tailed unpaired t-test with 95% confidence interval was used for statistical analysis. All statistical analysis, apart from the sequencing data analysis was carried out with GraphPad Prism 8 (RRID:SCR_002798) software (GraphPad, USA) (*=p≤0.05; **=p≤ 0.01; ***=p≤ 0.001, ****=p≤0.0001). Analyses for sequencing data were performed with R Project for Statistical Computing (RRID:SCR_001905) version 3.6.1 and Bioconductor (RRID:SCR_006442) version 3.9 unless otherwise stated. Error bars and n values used are described in the figure legends.

### RNA-seq of *in vitro* differentiated T-helper cells

RNA was extracted using the QIAShredder and RNeasy Mini Kit, or RNeasy Micro kit, both with on-column DNase digestion, according to the manufacturer’s instructions (Qiagen). RNA-seq libraries were made with total RNA equally pooled from the technical triplicate wells within an independent biological experiment, using the Illumina Stranded TruSeq Library preparation kit V2 and unique multiplexing indexes, according to the manufacturer’s instructions (Illumina). All libraries were then sequenced using the HiSeq 4000 system (Illumina) with paired-end read lengths of 100bp and at least 25 million reads per sample.

### ATAC-seq of *in vitro* differentiated T-helper cells

ATAC-seq samples from *in vitro* differentiated T-helper cells were prepared as outlined in 31. For each sample, 50,000 cells were lysed in cold lysis buffer containing 10mM Tris-HCl, pH 7.4, 10mM NaCl, 3mM MgCl2, 0.1% Nonidet™ P40 substitute (all Sigma) and the nuclei incubated for 2 hours at 37°C with 50μl of TDE1/TD transposase reaction mix (Illumina). Tagmented DNA was then purified using the MinElute kit (Qiagen) and amplified under standard ATAC PCR conditions: 72°C for 5 min; 98°C for 30s and thermocycling at 98°C for 10s, 63°C for 30s and 72°C for 1 min for 12 cycles. Each 50μl PCR reaction consisted of: 10μl Tagmented DNA, 10μl water, 25μl NEBNext High-Fidelity 2x PCR Master Mix (NEB), 2.5μl Nextera XT V2 i5 primer and 2.5μl Nextera XT V2 i7 primer (Illumina). NexteraXT V2 primers (Illumina) were used to allow larger scale multiplexing, these sequences were ordered directly from Sigma (0.2 scale, cartridge) and diluted to 100μM with 10mM Tris-EDTA buffer, pH8 (Sigma) and then to 25μM with DEPC-treated water (Ambion) for use in the reaction. Following amplification, ATAC-seq libraries were cleaned up using 90μl of AMPure XP beads (Beckman Coulter) and two 80% Ethanol washes whilst being placed on a magnetic plate stand, before being eluted in 1mM (0.1x) Tris-EDTA buffer, pH8 (Sigma) diluted with DEPC-treated water (Ambion). ATAC-seq libraries were then checked on the TapeStation/BioAnalyser (Agilent) before being sequenced on the HiSeq 4000 system (Illumina), with paired-end read lengths of 50bp and at least 50–80 million uniquely mapped reads per sample.

### RNA-seq: pre-processing and quality control ( *in vitro* CD4+ T cell datasets)

Paired end RNA-seq reads were quality controlled and adapters were trimmed using skewer (RRID:SCR_001151) software version 0.2.2 ^32^ with the following parameters: "-m pe -q 26 -Q 28 -e -l 30 -L 100", specifying the relevant adapter sequences. Reads were then aligned to mm10 genome and the GENCODE reference transcriptome version M22 using STAR (RRID:SCR_004463) software version 2.7.1 ^33^, excluding multi-mapping reads by setting the parameter “outFilterMultimapNmax” to 1. In order to increase read mapping to novel junctions the parameter “twopassMode” was set to "Basic". Raw gene counts were retrieved using QoRTs software version 1.1.8 ^34^, specifying the “stranded” parameter for the *in vitro* CD4 ^+^T cell datasets due to the nature of the library preparation. Normalized read counts were retrieved using DESeq2 (RRID:SCR_015687) version 1.24.0 ^35^ and rlog transformed in order to visualize gene quantifications.

### RNA-seq kinetics analysis of *in vitro* CD4+ T cells

Differentially expressed genes (DEG) at any given time point for IL-12, IL-12+IL-27, and IL-27 compared to medium control were obtained using DESeq2 (fold change >=1.5 and Benjamini-Hochberg adjusted p value<0.05), resulting in a total of 2,300 DEG. Next, the gene expression values of these DEG were subjected to *k-*means clustering using a *k*=9; where the optimal *k* was obtained using the R library “factoextra” (RRID:SCR_016692) (factoextra 2017). The expression values of the 2,300 DEG were standardized per gene (row z score) and plotted in a heatmap. The mean expression and 90% c.i. were obtained for these clusters and plotted.

### Transcription factors correlating with *Il10* expression

A gene was considered to be coding for a transcription factor if it was present in at least two of the following references ^36– 38^, or Ingenuity Pathway Analysis (RRID:SCR_008653) (IPA) database (genes annotated as either "transcription regulator" or "ligand-dependent nuclear receptor") (QIAGEN Redwood City, www.qiagen.com/ingenuity).

Genes encoding for transcription factors that have expression patterns correlating with *Il10* gene expression were analysed (absolute Pearson's correlation >0.7) from Days 1 to 4 in all conditions. A linear regression model was fitted for the top 9 transcription factors positively correlating with *Il10* expression.

### Genome-wide differential footprint detection with BaGFoot software

To identify potential transcription factors underlying the gene expression changes occurring between Day 2 and Day 3, we applied the BaGFoot software on our ATAC-seq data ^39^, using all ATAC-seq peaks identified in each treatment condition at Day 2 and Day 3. BaGFoot predicts these changes by searching for TF-binding motif matches in regions with altered ATAC-seq insertion patterns between the two days. We used 318 motifs of class A and B quality in the HOCOMOCO (RRID:SCR_005409) database v11 ^40^. BaGFoot does not consider replicates for the analysis, thus we performed two pair-wise comparisons (using all biological replicates) for each condition and calculated the average changes in accessibility and footprint-depth. Results are displayed as bagplots, using a fence of factor 2. We identified TFs with potentially altered binding between Day 2 and Day 3 by identifying the outliers of the multivariate distribution, as assessed by the Mahalanobis distance of each TF to the multivariate distribution ^39^. The statistical significance of these distances was tested using a Chi-square distribution followed by a Benjamini-Hochberg correction for multiple-testing, as recommended by BaGFoot, and shown as tables.

### Differential gene expression analysis of *in vitro* CD4 ^+^ T cells: *Cd4* ^Cre^-mediated deletion of transcription factors versus floxed controls

The DEG of CD4 ^+^ T cells with *Cd4* ^Cre^-mediated deletion of *Prdm1*, *Maf*, or both *Prdm1* and *Maf* against their corresponding floxed controls were obtained for all conditions (Medium, IL-12, IL-12+IL-27, and IL-27) using DeSeq2 with the default thresholds (Benjamini-Hochberg adjusted p value<0.1), this resulted in 208, 561, and 802 for cells with *Cd4* ^Cre^-mediated deletion of *Prdm1*, *Maf*, and both *Prdm1* and *Maf*, respective.

### Singular Value Decomposition analysis and biological interpretation

Singular Value Decomposition (SVD) analysis was performed as in 16 on each set of samples that shared a genetic background: a) *Prdm1* ^fl/fl^ *Cd4* ^Cre^ was analysed with *Prdm1* ^fl/fl^, b) *Maf* ^fl/fl^ *Cd4* ^Cre^ with *Maf* ^fl/fl^, and c) *Prdm1* ^fl/fl^ *Maf* ^fl/fl^ *Cd4* ^Cre^ with *Prdm1* ^fl/fl^ *Maf* ^fl/fl^. Prior to the SVD analysis, the rlog-normalized gene counts were "centered" by subtracting the mean expression per gene. The average values of the right-singular vectors, which relate the association of each sample to a component, were plotted as bar-plots.

For each right-singular vector three linear models were fitted: 1) a full linear model containing the variables for the differentiation condition and the genotype, 2) a reduced model containing only the differentiation condition, and 3) a reduced model containing only the genotype. In order to identify the association of each component with the condition and/or genotype the Akaike Information Criterion (AIC) score was calculated and an analysis of variance (ANOVA) with a Chi-squared-test was performed between the full model (1) versus the reduced ones (2 and 3). The component capturing the *Cd4* ^Cre^-mediated transcription factors deletion was chosen using the following criteria:

a) The component where the AIC of the reduced genotype model is lower than AIC of the full mode (line-plot)
b) The component with the smallest p value in the ANOVA (heatmap). Only statistically significant values (BH adjusted p value <0.05) were plotted for visualisation.
c) The component in which the average values of the right-singular vectors diverge in sign for *Cd4* ^Cre^-mediated transcription factors deletion *vs.* floxed controls shown as histograms.

The left-singular vectors, which relate the contribution of a gene to a component, were segregated between positive and negative values, and each set was subjected to *k-*means clustering ( *k*=2). The genes belonging to the most positive and negative clusters were selected for further examination and they are referred in the text as the "SVD components associated with *Cd4* ^Cre^-mediated deletion".

For each genetic background the standardised expression values (row z score) of genes belonging to the component capturing the *Cd4* ^Cre^-mediated transcription factors deletion are shown in the heatmaps.

To further dissect the genes mostly affected by the *Cd4* ^Cre^-mediated transcription factors deletion a matrix containing the fold-changes of the three genetic backgrounds ( *Prdm1*, *Maf*, and *Prdm1xMaf*) was created and subjected to *K* means clustering (k=7).

### Data annotation

Gene Ontology (GO) ^41, 42^ (RRID:SCR_002811) enrichment was assessed using the R package “topGO” (RRID:SCR_014798) ^43^. The top 100 GO terms of "biological processes'' were further synthesized using REViGO (RRID:SCR_005825) ^44^ with allowed similarity=0.4. The top 10 GO terms were shown for annotation.

### ATAC-seq: pre-processing and quality control of *in vitro* CD4+ T cells

Paired end ATAC-seq reads were quality controlled and adapters were trimmed using Skewer software version 0.2.2 ^32^ with the following parameters: "-m pe -q 26 -Q 30 -e -l 30 -L 50", specifying "CTGTCTCTTATACAC" as reference adapter sequence to remove. Quality controlled reads were then aligned to mm10 genome using BWA-MEM (RRID:SCR_010910) ^45^ with (Picard toolkit 2018 (RRID:SCR_006525)) and SAMtools (RRID:SCR_002105) 1.3.1 ^46^ was used to discard discordant alignments and/or with low mapping qualities (mapQ<30). In order to account for transposase insertion, reads were shifted +4bp in the forward and -5bp in the reverse strand; moreover, read-pairs that spanned >99bp were excluded from further analyses as they would span nucleosomes ^31^.

### Identification of open chromatin sites

MACS2 (version 2.1.1) (RRID:SCR_013291) was used to identify ATAC-seq peaks using the following parameters: "parameters --keep-dup all --nomodel --shift -100 --extsize 200; q-value < 0.01", in order to identify enrichment of Tn5 cutting sites ^47^.

### Differentially accessible site detection *in vitro* CD4+ T cells

In order to identify open chromatin sites that differed in accessibility between *Cd4* ^Cre^-mediated transcription factor deletion and floxed controls, DiffBind (RRID:SCR_012918) software version 2.0.2 ^48^ was used with the following parameters: "dba.count:minOverlap=0, score= DBA_SCORE_RPKM, bRemoveDuplicates=FALSE, bUseSummarizeOverlaps= TRUE; dba.analyze: method=DBA_DESEQ2, bFullLibrarySize=T" for each condition and genetic background. An ATAC-seq peak was considered to represent remodelled chromatin if the absolute fold-change>1.5 and FDR<0.05.

### Identification of c-Maf and Blimp-1 putative binding sites

c-Maf ChIP-seq raw fastq files were obtained from GSE40918 ^20^ and Blimp-1 ChIP-seq raw fastq files were obtained from GSE79339 ^49^ and GSE66069 ^50^. Trimmomatic version 0.36 was used for quality control and trim adapter sequences using the following parameters: "HEADCROP:2 TRAILING:25 MINLEN:26" ^51^. Trimmed reads were aligned to mouse genome mm10 with Bowtie (RRID:SCR_005476) 1.1.2 ^52^ with the parameters: "y -m2 --best --strata -S". MACS2 2.1.1 was used with default parameters to identify ChIP-seq peaks, and peaks with a q-value<0.01 were defined as statistically significant binding sites. For each transcription factor, a final peak set was generated from the union of the statistically significant binding sites identified in each biological replicate (c-Maf) or GEO dataset (Blimp-1). This resulted in 45,727 ChIP-seq binding sites for c-Maf and 16,893 binding sites for Blimp-1. CRUNCH suite ^53^ was used to infer the c-Maf motif, as the source dataset provided biological replicates and a suitable input control. On the other hand, the motif of Blimp-1 was taken from HOCOMOCO database v11 ^40^ as two distinct ChIP-seq datasets were used, neither with biological replicates, thus not suitable for analysis with CRUNCH. These identified motifs were used as input for FIMO software ^54^ the sequences underlying the ATAC-seq peaks were scanned for motif-matches in order to identify further putative binding sites of c-Maf and Blimp-1.

### Visualisation of genome browser tracks

"bamCoverage" from DeepTools (RRID:SCR_016366) 2.4.2 was used to normalize ATAC-seq data to RPKMs and the R package "ggbio" (RRID:SCR_003313) was used to visualize the genome browser tracks ^55^. The CNS sites marked have the following coordinates ( Table 1).

**Table 1.** Coordinates correspond to mm10 genome.

Gene	Chromosome	start	end	CNS	Citation
*Il10*	1	130999687	130999976	-20	18
*Il10*	1	131010529	131010907	-9	18
*Il10*	1	131015400	131015593	-4.5	18
*Il10*	1	131019438	131019696	-0.5	18
*Il10*	1	131025957	131026170	6.45	57
*Prdm1*	10	44444389	44444891	14	18
*Prdm1*	10	44459445	44459716	-1	18
*Prdm1*	10	44459873	44460126	-1.5	18
*Prdm1*	10	44460425	44460609	2	18
*Ifng*	10	118435228	118435850	-6	58
*Ifng*	10	118419035	118419610	-22	58
*Ifng*	10	118406839	118407520	-34	58
*Ifng*	10	118458481	118459017	18	58
*Ifng*	10	118460275	118460853	20	58
*Ifng*	10	118470550	118471067	29	58
*Maf*	8	115707132	115707487	-0.5	59
*Maf*	8	115707694	115708065	-1	59

Integration of ATAC-seq, ChIP-seq, motifs, and RNA-seq from *in vitro* CD4+ T cells. ChIP-seq-identified binding sites of c-Maf and Blimp-1 were filtered using the ATAC-seq peaks in order to obtain those binding sites that were biologically relevant to our *in vitro* CD4 ^+^ T cells datasets. At this stage, each ATAC-seq had assigned an overlapping ChIP-seq peak, a motif, or none. A gene was assigned to an ATAC-seq peak based on distance proximity, if the peak was within +/- 3kb of the gene body coordinates, using the R package “ChIPSeeker” (RRID:SCR_021322) ^56^. Thus, a gene was a direct target of c-Maf and/or Blimp-1 if said gene was annotated with an ATAC-seq peak containing a putative binding site from either transcription factor (identified by motif or ChIP-seq). To quantitatively rank abundance of c-Maf or Blimp-1 binding sites in each gene, we applied the same approach as before in 16, additionally the likelihood of c-Maf or Blimp-1 to regulate a gene was calculated using the Binding and Expression Target Analysis (BETA) (RRID:SCR_005396) software, with the following parameters: "plus -g mm10 --da 0.5 --df 1 -c 1".

Direct targets of c-Maf and/or Blimp-1 are depicted in the gene regulatory networks. For all networks, *Maf* and *Prdm1* nodes were added, in order to visualize target genes of Blimp-1 and c-Maf. These gene regulatory networks show the integration of all these "omic" datasets and were generated using the "igraph" R package ^60^. Each node represents a gene, and the size of a node represents the left-singular vectors obtained from the SVD analysis, thus relating the effect on expression the *Cd4* ^Cre^-mediated deletion of *Prdm1* and/or *Maf* had on a gene. The edges depict the relationship of a target gene with c-Maf and Blimp-1, the thickness of the edge shows the likelihood of a gene being a target of either c-Maf or Blimp-1 as assessed by the BETA software. The colouring of the edge shows if the target gene has a c-Maf (green), Blimp-1 (pink), or c-Maf and Blimp-1 (blue) binding site assigned. For visualization purposes, the size of the maximum left-singular vector was fixed to be equal to the 2 ^nd^ highest; additionally, the labels of gene names were only added for the 50 most affected genes according to the SVD analysis. Scores used to generate the networks are available in Supplementary Table 7 as *Underlying data* ^30^.

## Results

### Expression of *Prdm1* and *Maf* correlates with *Il10* expression in Th1 cells

**Figure 1.**
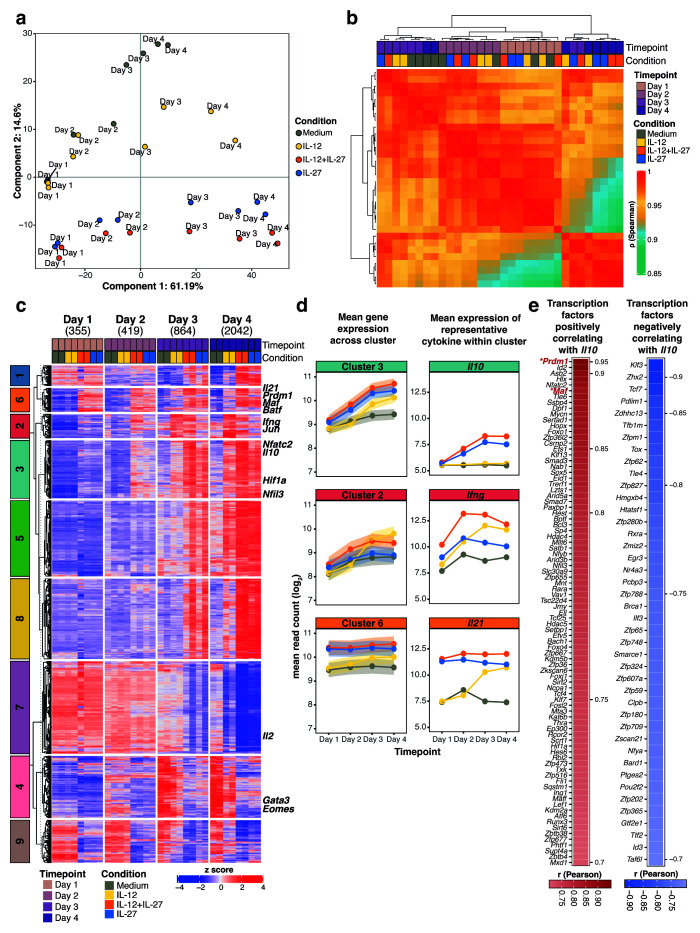
Temporal gene regulation by IL-27 in CD4 ^+^ T cells is accompanied by clusters of candidate transcription factors that correlate with *Il10* expression, including Blimp-1 and c-Maf. RNA-seq analysis of flow cytometry sorted naïve CD4 ^+^ T cells activated *in vitro* with anti-CD3 and anti-CD28 antibodies and differentiated in the presence of Medium (no cytokines), IL-12, IL-12+IL-27, or IL-27 from Day 1 to Day 4. **a**, Principal component analysis (PCA) showing the two most dominant variables, time point (PC1: Day 1-2 versus Day 3-4) and differentiation condition (PC2: presence versus absence of IL-27 during differentiation). **b**, Unsupervised hierarchical clustering of a pair-wise Spearman correlation of samples encompassing CD4 ^+^ T cells differentiated *in vitro* in the presence of Medium, IL-12, IL-12+IL-27, or IL-27 from Day 1 to Day 4. **c**, Heatmap visualization of differentially expressed genes per condition compared to Medium (fold change >=1.5 and BH adjusted p value<0.05), partitioned into 9 clusters using *k*-means clustering. The values above the heatmap between parentheses show the number of differentially expressed genes at each time point. **d**, Gene expression profiles depicting the mean gene expression ±90% confidence intervals (c.i.) across all genes for clusters 3, 2 and 6 accompanied by mean gene expression for representative cytokines *Il10*, *Ifng* and *Il21* respectively. **e**, All transcription factors annotated in the mouse genome positively correlating (Pearson’s *r*>0.7) and negatively correlating (Pearson’s *r*<0.7) with *Il10* expression in *in vitro* CD4 ^+^ T cells differentiated in the presence of Medium, IL-12, IL-12+IL-27, or IL-27 from Day 1 to Day 4. Data from n=2 biological replicates.

We previously showed that T cell-specific deletion of *Maf* resulted in the maximal reduction of IL-10 production by Th1 cells *in vivo* as compared to other T cell subsets ^16^. However, since this effect was incomplete, we set out to identify additional transcription factors that positively regulate *Il10* in Th1 cells. To achieve this, we first analysed changes in temporal gene expression *in vitro* in naïve CD4 ^+^ T cells stimulated with anti-CD3 and anti-CD28 as described in the Methods, and differentiated over time into Th1 cells with either IL-12 or IL-12 plus IL-27 (IL-12+IL-27), as compared to IL-27 alone (referred to as ‘Tr1 cells’ by Zhang *et al.*, ^13^), or medium as controls. The combination of IL-12 and IL-27 has been shown *in vivo* to be required for maximal levels of IFN-γ and IL-10 production by CD4 ^+^ Th1 cells ^12, 14, 15^. Cells were cultured under these different conditions for 4 days and sampled at each time point for RNA-based next-generation sequencing (RNA-seq). Cells clustered distinctly for the most part according to time point of differentiation ( Figure 1a, Figure 1b), with the principal component 1 separating days 1 and 2 from days 3 and 4 ( Figure 1b; Supplementary Table 1 in *Underlying data* ^30^).

Co-regulated clusters of gene expression were revealed using k-means clustering ( Figure 1c; Supplementary Table 2 in *Underlying data* ^30^). Clusters 3, 2 and 6 each expressed effector T cell cytokines genes, including *Il10, Ifng and Il21*, respectively ( Figure 1c). Expression of *Il10* (Cluster 3) was seen in IL-12+IL-27 or IL-27 driven T cells, peaking at days 3 and 4 of culture, although other genes within this Cluster 3 were also increased similarly in Th1 cells driven by IL-12 alone ( Figure 1c and Figure 1d). By contrast, expression of *Ifng* within Cluster 2, was maximal in IL-12+IL-27 driven Th1 cells from days 2–4 of culture. *Ifng* showed delayed induction in IL-12 alone driven Th1 cells, and a small increase in IL-27 alone driven T cells by day 2, which then decreased with time to the levels seen in medium control cultures ( Figure 1c and d). Expression of *Ifng c*lustered with expression of the transcription factor *Jun* ( Figure 1c). Collectively the expression of the genes in Cluster 6 was induced by IL-12+IL-27 and IL-27 alone and to a lesser extent in IL-12 driven Th1 cells and was maximal from days 1–4 of culture ( Figure 1c and d). Expression of *Il21* was also observed within this Cluster 6 and was maximally induced in IL-12 + IL-27 and IL-27 alone driven T cells by day 1 to day 4 of culture, however IL-12 driven Th1 cells only started to express *Il21* by days 3 and 4 of culture as compared to medium controls ( Figure 1c and d). Our findings suggest a role for IL-21 in autocrine expansion of T effectors rather than as a regulator of *Il10* as has been previously suggested ^61, 62^, since it is produced maximally by Th1 cells, which do not produce IL-10.

**Figure 2.**
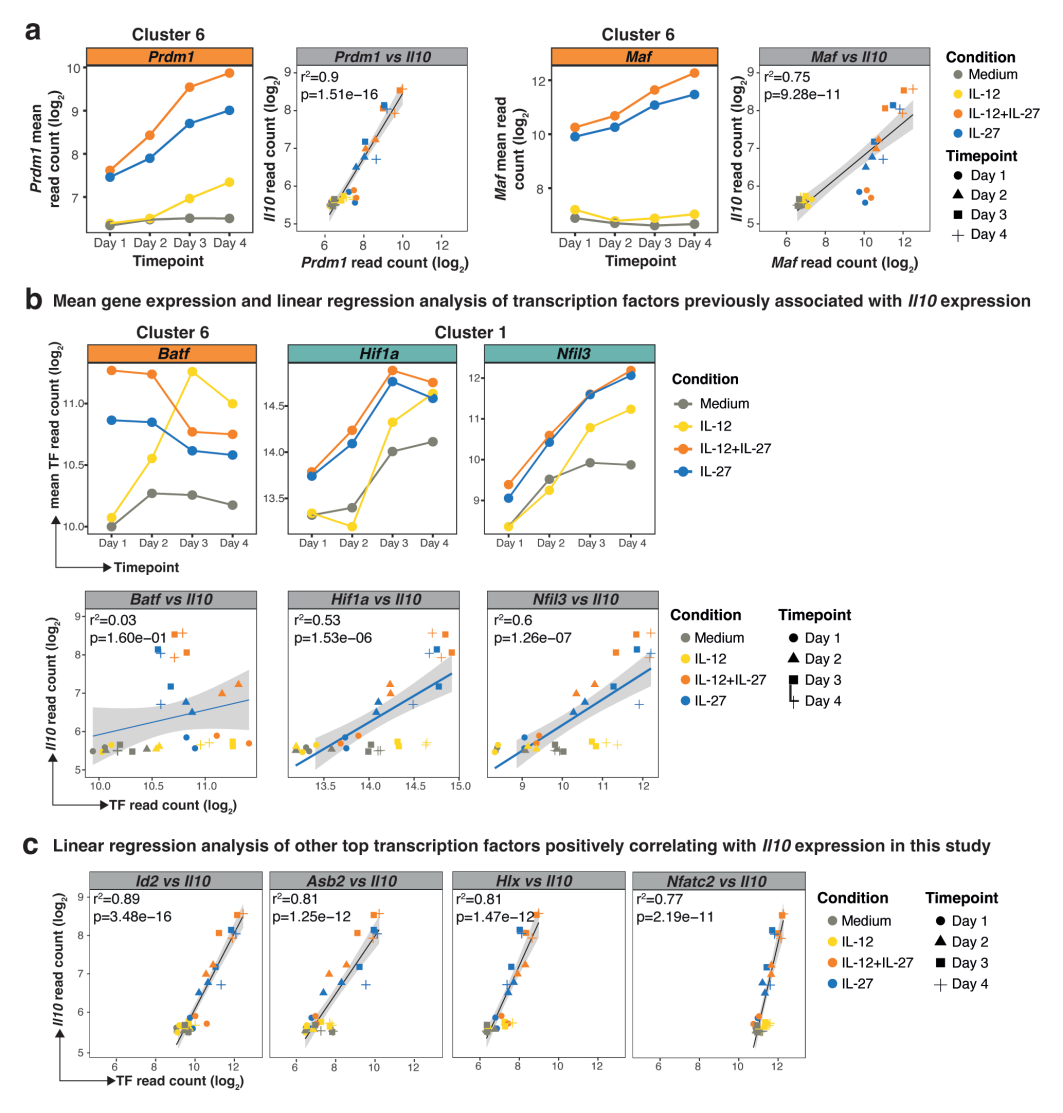
Blimp-1 and c-Maf strongly positively correlate with IL-27 induced *Il10* expression in differentiating naïve CD4 ^+^ T cells. RNA-seq analysis of flow cytometry sorted naïve CD4 ^+^ T cells activated *in vitro* with anti-CD3 and anti-CD28 antibodies and differentiated in the presence of Medium (no cytokines), IL-12, IL-12+IL-27, or IL-27 from Day 1 to Day 4. **a**, Mean gene expression of *Prdm1* and *Maf,* both transcription factors positively correlating with *Il10* expression, accompanied by linear regression of *Prdm1* and *Maf* against *Il10* expression across all conditions and timepoints. **b**, Mean gene expression profiles (top panel) of transcription factors previously associated with *Il10* expression. Linear regression (bottom panel) of these transcription factors against *Il10* expression across all conditions and timepoints. **c**, Linear regression of additional top 4 positively correlating transcription factors against *Il10* expression identified in this study across all conditions and timepoints. Data from n=2 biological replicates.

*Il10* expression clustered with the transcription factors *Nfatc2, Hif1a* and *Nfil3* (Cluster 3), while *Il21* expression clustered with the transcription factors *Prdm1, Maf* and *Batf* (Cluster 6), all of which were highly expressed upon culture with IL-12+IL-27 or IL-27 alone ( Figure 1c). Regardless, the transcription factors showing the highest positive correlation with *Il10* expression were first *Prdm1*, then *Id2, Asb2, Hlx, Nfatc2* and *Maf* ( Figure 1e). Expression of *Prdm1* and *Maf* was observed by day 1 but was maximal at days 3 and 4 of culture in IL-12+IL-27 and IL-27 alone ( Figure 2a), coinciding and correlating with the peak of *Il10* gene expression ( Figure 1d and Figure 1e), and correlating significantly with *Il10* expression ( Figure 2a). However, expression of *Batf* under these conditions was maximal on days 1 and 2, but rapidly diminished by days 3 and 4, while increasing with time in IL-12 alone driven cultures and did not show a good correlation with *Il10* expression ( Figure 2b), suggesting a broader function than the regulation of *Il10* ( Figure 2b). Other transcription factors that have been associated with *Il10* gene expression include *Hif1a* and *Nfil3* ^13, 16^ only showed a slight correlation with *Il10* expression ( Figure 2b). By contrast, the other transcription factors *Id2, Asb2, Hlx* and *Nfatc2* that correlated with *Il10* gene expression ( Figure 1e) showed a high level of significance ( Figure 2c), in keeping with the literature ^13^.

**Figure 3.**
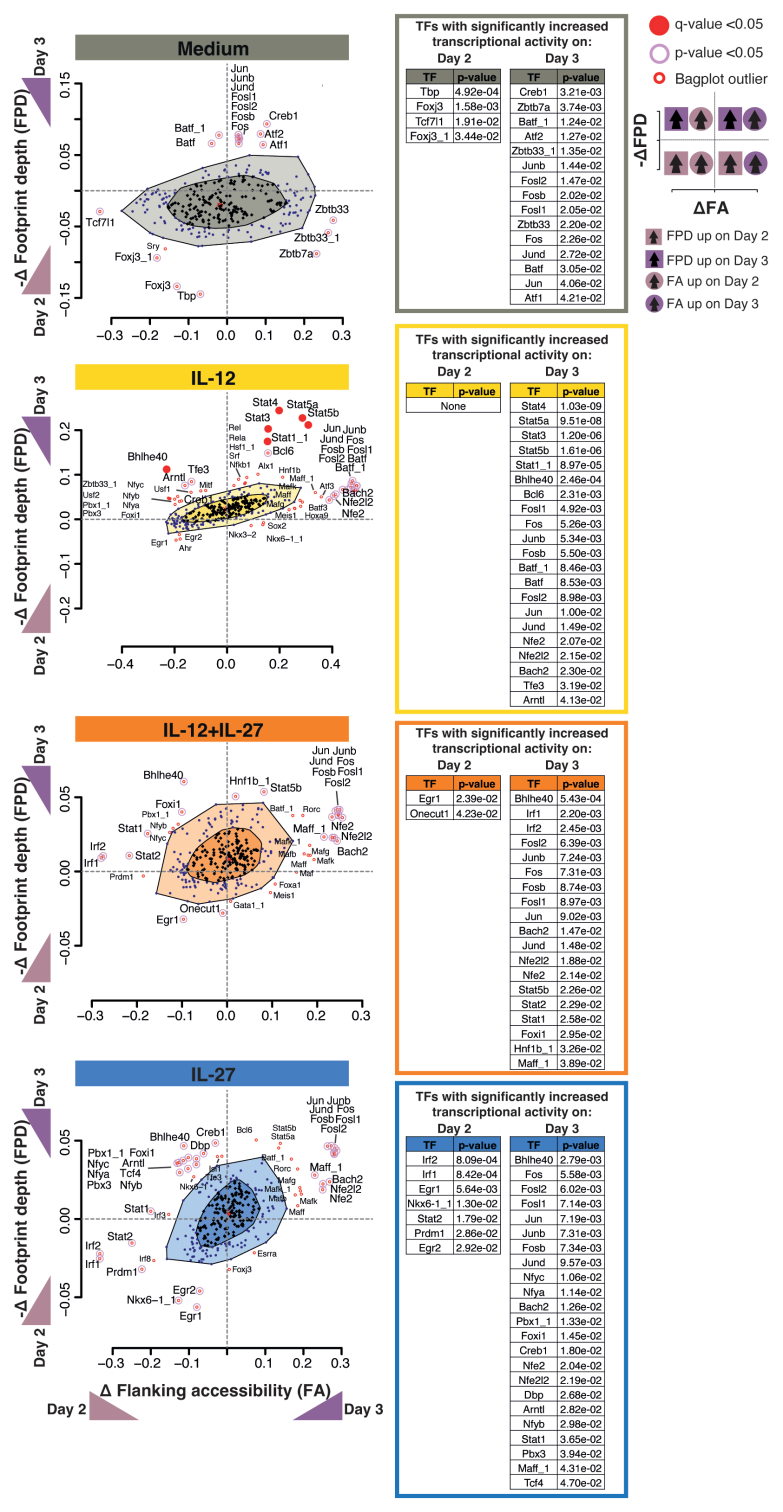
Blimp-1 and c-Maf have increased differential binding between Day 2 and Day 3 of culture only under IL-12+IL+27 and IL-27 cytokine driving conditions. BaGFoot analysis of transcription factors with putative genome-wide changes in chromatin binding between Day 2 and Day 3, as assessed by Tn5 insertion patterns (obtained with ATAC-seq) in *in vitro* activated and differentiated CD4 ^+^ T cells in the presence of Medium (no cytokines), IL-12, IL-12+IL-27, or IL-27. Presented as the change in change in the ‘flanking accessibility’ of motifs (ΔFA) plotted against the ‘footprint depth’ (–ΔFPD); wedges along axes indicate direction and degree of change in transcription factor binding between Day 2 and Day3. Dark shading in the “bagplots” indicates a region with no change in transcription factor binding patterns, and light shading indicates a region in which most non-significant minor changes in binding occur. Transcription factors with significant change in binding are found as outliers outside the “bagplot”. A table of P values is provided for the outliers of the “bagplot” and indicates the statistical confidence assigned to the differential binding between Day 2 and Day 3 of a transcription factor in each CD4 ^+^ T cell differentiation condition.

To further investigate global changes in transcriptional activity in CD4 ^+^ naïve T cells cultured as above, we used the assay for transposase-accessible chromatin plus sequencing (ATAC-seq) to reveal functionally active genomic regions at days 2 and 3, timepoints, which marked key transcriptional changes in the differentiation of CD4 ^+^ naïve T cells ( Figure 1a). The ‘bivariate genomic footprinting’ (BaGFoot) software ^39^ was applied to the ATAC-seq data, to detect transcriptional activity changes occurring between day 2 and day 3, as assessed by differences in transcription factor ‘footprint depth’ and ‘flanking accessibility’ of the underlying transcription factor motifs. IL-27 and IL-12+IL-27 cultured T cells showed increased transcriptional activity for *Prdm1* and *Maf*, whereas transcriptional activity of the AP-1 family member, *Batf,* was only evident in cells cultured in medium alone or IL-12; and *Stat 3, 4* and *5* only under IL-12 conditions. Other AP-1 family members *Jun* and *Fos* showing increased transcriptional activity across all conditions ( Figure 3).

### Reduction or abrogation of *Il10* expression in IL-12+IL-27 and IL-27-driven CD4+ T cells upon deletion of *Prdm1, Maf* or both *Prdm1* and *Maf*, is accompanied by increased *Ifng* expression

**Figure 4.**
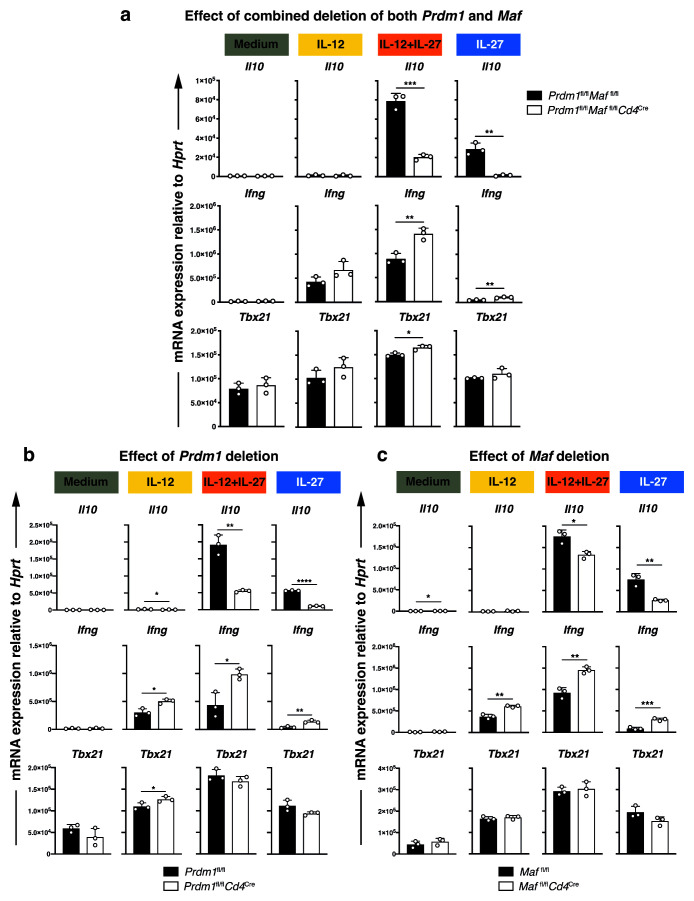
Blimp-1 and c-Maf promote the expression *Il10*, whilst repressing *Ifng* in CD4 ^+^ T cells without affecting differentiation capacity in presence of IL-12, IL-12+IL-27, or IL-27. Naïve CD4 ^+^ T cells activated *in vitro* with anti-CD3 and anti-CD28 antibodies and differentiated in the presence of Medium (no cytokines), IL-12, IL-12+IL-27, or IL-27 to Day 3. **a**– **c**, Real-time quantitative PCR analysis of *Il10, Ifng* and *Tbx21* expression upon *Cd4* ^Cre^-mediated deletion of **a**, both *Prdm1* and *Maf*, and either **b**, *Prdm1* or **c**, *Maf* alone. Graphs show mean of technical triplicates ±SD. Data representative of n=3 independent biological experiments.

Since expression of *Il10* in IL-12+IL-27 and IL-27-driven CD4 ^+^ T cells appeared to correlate strongly with expression of *Prdm1* as well as *Maf* over time, while *Ifng* expression appeared to be repressed at peak times under these conditions, we wished to determine the requirement of *Prdm1* and/or *Maf* in the regulation of both cytokines. To address this, naïve CD4 ^+^ T cells from *Prdm1* ^fl/fl^ *Cd4* ^Cre^, *Maf* ^fl/fl^ *Cd4* ^Cre^, *Prdm1* ^fl/fl^ *Maf* ^fl/fl^ *Cd4* ^Cre^ and respective floxed control mice, were differentiated into Th1 cells, with IL-12 or IL-12+IL-27, and IL-27 alone or medium controls (as in Figure 1) and expression of *Il10, Ifng* and *Tbx21* was first assessed by RT-PCR ( Figure 4). Th1 cells differentiated with IL-12+IL-27 and IL-27-driven T cells showed significant levels of *Il10* expression, which was diminished in the absence of *Prdm1* or *Maf*, and to the greatest extent in the absence of both transcription factors ( Figure 4a–c). Effects on T cells differentiated with IL-27 alone are in keeping with findings of Zhang *et al.*, in ‘Tr1 cells’ ^13^. In addition to the effects that we observed on *Il10* expression in the IL-12+IL-27-driven Th1 cells, the absence of *Prdm1, Maf* or both transcription factors, resulted in an increase in *Ifng* expression, while the expression of the Th1/IFN-specific transcription factor *Tbx21* ^63^ was not significantly affected ( Figure 4a–c).

### Deciphering the transcriptional programs regulated by Blimp-1 and c-Maf in CD4 ^+^ T cells

**Figure 5.**
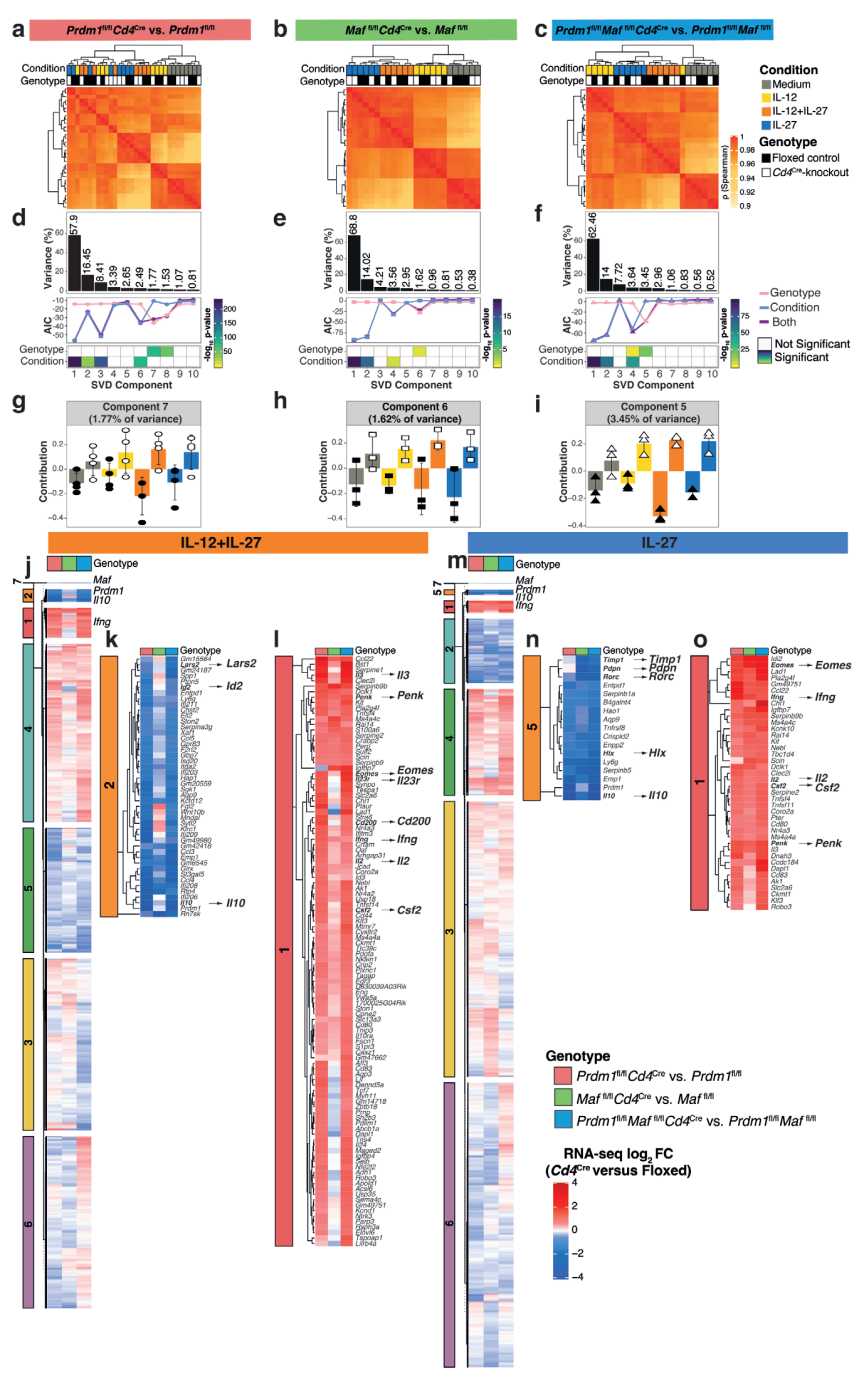
Deciphering the transcriptional programs regulated by Blimp-1 and c-Maf in CD4 ^+^ T cells. RNA-seq analysis of CD4+ T cells differentiated *in vitro* with Medium, IL-12, IL-12+IL-27, or IL-27 on Day 3 with *Cd4* ^Cre^-mediated transcription factor deletion. **a**– **c**, Clustering of Spearman correlation showing the effect of deletion of **a**, *Prdm1*, **b**, *Maf*, or **c**, both *Prdm1* and *Maf* on gene expression across all conditions. **d**– **f**, Singular value decomposition (SVD) analysis identifying changes in gene expression upon deletion of **d**, *Prdm1*, **e**, *Maf*, or **f**, both *Prdm1* and *Maf*. Histograms depict percentage of variance explained for the first ten SVD-components. To statistically determine the variables associated with each component, the Akaike information criterion (AIC) score (line-plot) and ANOVA p values ( *X* ^2^ test; heatmap) were calculated. **g**– **i**, Average values ±SD for the SVD right-singular vectors of each component capturing *Cd4* ^Cre^-mediated transcription factor deletion, with dots representing individual samples. **j** and **m** Heatmap of fold-changes for genes within the SVD components associated with *Cd4* ^Cre^-mediated transcription factor deletion in cells differentiated with **j**, IL-12+IL-27 and **m**, IL-27 alone. Fold-change values were subjected to *k-*means clustering to unbiasedly identify genes most affected. **k**– **l** and **n**– **o** Heatmap of genes most affected by *Cd4* ^Cre^-mediated transcription factor deletion in **k**– **l** IL-12+IL-27 and **n**– **o**, IL-27 alone differentiated cells (extracted from heatmaps in **j** and **m**). Data from n=3–4 biological replicates.

**Figure 6.**
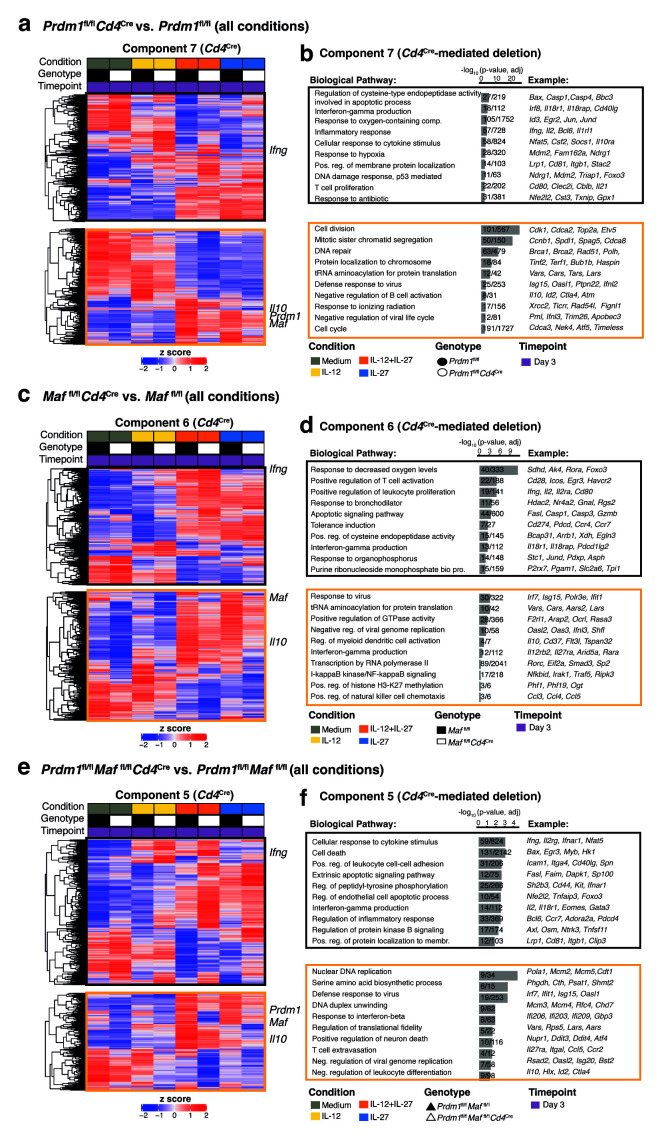
Deciphering the transcriptional programs regulated by Blimp-1 and c-Maf in *in vitro* differentiated CD4+ T cells from dominant IL-27 and IL-12 driven transcriptional changes. **a**, **c**, **e**, For each SVD component associated with *Cd4* ^Cre^-mediated deletion of either **a**, *Prdm1*, **c**, *Maf,* or **e**, both *Prdm1* and *Maf* contributing genes were partitioned into “positively associated” (black outline) or “negatively associated” (orange outline) with the SVD component and their expression values visualized in a heatmap. **b**, **d**, **f**, Gene ontology biological pathways enriched within the SVD component associated with *Cd4* ^Cre^-mediated deletion of either **b**, *Prdm1*, **d**, *Maf,* or **f**, both *Prdm1* and *Maf.* Data from n=3–4 biological replicates.

To determine the role of Blimp-1 and c-Maf on the regulation of cytokine gene networks in an unbiased fashion, naïve CD4 ^+^ T cells from *Prdm1* ^fl/fl^ *Cd4* ^Cre^, *Maf* ^fl/fl^ *Cd4* ^Cre^, *Prdm1* ^fl/fl^ *Maf* ^fl/fl^ *Cd4* ^Cre^ and respective floxed control mice, were differentiated with IL-12 or IL-12+IL-27, and IL-27 or medium controls as described for Figure 1 and Figure 4. Cells were sampled at day 3 and processed for RNA-seq, which was then subjected to bioinformatics analyses ( Figure 5; Supplementary Table 4 in *Underlying data* ^30^). Hierarchical clustering of a Pearson’s correlation analyses in each genotype revealed that the greatest variations in gene expression were cytokine driven, while differences resulting from transcription factor deletion were difficult to discern ( Figure 5a–c). Variance captured by singular-value-decomposition (SVD) components ( Figure 5d–i) allowed clustering of differential gene expression according to the cytokine-driven conditions and additionally the effects of transcription factor deletion, supported by biological pathway analysis ( Figure 5 and Figure 6; Supplementary Table 5 in *Underlying data* ^30^). The variance explained by the SVD components capturing the *Cd4* ^Cre^-mediated transcription factors deletion were: 1.77% for the *Prdm1* ^fl/fl^ *Cd4* ^Cre^ (Component 7); 1.62% for the *Maf* ^fl/fl^ *Cd4* ^Cre^ (Component 6); and 3.45% for the *Prdm1* ^fl/fl^ *Maf* ^fl/fl^ *Cd4* ^Cre^ (Component 5) ( Figure 5d–f), with average values for the SVD right-singular vectors of each component capturing *Cd4* ^Cre^-mediated transcription factor deletion shown in Figure 5g–i. It is of note, that while deletion of *Prdm1* and *Maf* in CD4 ^+^ T cells demonstrated the effects of Blimp-1 and c-Maf in regulating *Il10* and negatively regulating common and unique proinflammatory gene networks in IL-12+IL-27-driven Th1 cells and IL-27 driven “Tr1” cells, these effects were less pronounced than gene expression changes resulting from TCR-stimulation and culture in the cytokines, IL-12, IL-12+IL-27, or IL-27 ( Figure 5d–f, Component 1, which constitute 58-69% of the response) and ( Figure 5d–f, Component 2 or Component 3, which constitute 14-16% or 4-8% of the response, respectively).

Heatmap values of fold-changes for genes within the combined SVD components associated with *Cd4* ^Cre^-mediated transcription factor deletion in cells differentiated with IL-12+IL-27 or IL-27 were subjected to *k-*means clustering to identify clusters of genes that were most affected by the different transcription factors in an unbiased fashion ( Figure 5j and 5m; Supplementary Table 6 in *Underlying data* ^30^). The average fold-changes for each cluster showed *k-*means Cluster 2 to be the most decreased, and Cluster 1 the most increased in *Prdm1* ^fl/fl^ *Cd4* ^Cre^ and *Prdm1* ^fl/fl^ *Maf* ^fl/fl^ *Cd4* ^Cre^, but less so in *Maf* ^fl/fl^ *Cd4* ^Cre^ T cells, as compared to floxed controls, when differentiated with IL-12+IL-27 ( Figure 5j–l). Cluster 2 contained the genes most decreased in expression including *Il10* ( Figure 5k). Conversely, Cluster 1, contained a large number of proinflammatory effector genes that were the most increased in expression including, *ll3, Penk, Eomes, Il23r, Cd200, Ifng, Il2* and *Csf2* ( Figure 5l). When differentiated with IL-27 alone the average fold-changes in the T cells for each cluster showed *k-*means Cluster 5 to be the most decreased, and Cluster 1 the most increased, in all transcription factor deficient T cells including *Prdm1* ^fl/fl^ *Cd4* ^Cre^, *Maf* ^fl/fl^ *Cd4* ^Cre^ and *Prdm1* ^fl/fl^ *Maf* ^fl/fl^ *Cd4* ^Cre^, as compared to floxed controls ( Figure 5m). Cluster 5, containing the genes most decreased in expression included *Il10, Timp1, Pdpn, Rorc* and *Hlx* ( Figure 5n). Conversely, Cluster 1, containing proinflammatory effector genes that were the most increased in expression included, *Eomes, Ifng, Il2, Csf2 and Penk* ( Figure 5o), suggesting that in addition to upregulating *Il10,* Blimp-1 *(Prdm1)* and c-Maf may directly repress proinflammatory genes to reinforce a suppressive response.

GO enrichment analysis was also applied to each SVD component associated with *Cd4* ^Cre^-mediated deletion of either *Prdm1*, *Maf,* or both *Prdm1* and *Maf* ( Figure 6a–f). Contributing genes were partitioned into “positively associated” (black outline) or “negatively associated” (orange outline) with the SVD component and their expression values visualized in a heatmap ( Figure 6a, 6c and 6e) and annotated using the biological processes within the GO database ( Figure 6b, 6d and 6f). An increase in expression of *Ifng* and Th1-associated and T cell activation-associated pathways was observed across all cytokine-driven conditions even in the absence of detectable *Il10* expression, such as in Th1 cells driven by IL-12 alone, and was the most pronounced in *Prdm1* ^fl/fl^ *Cd4* ^Cre^ and *Prdm1* ^fl/fl^ *Maf* ^fl/fl^ *Cd4* ^Cre^ but less so in *Maf* ^fl/fl^ *Cd4* ^Cre^ T cells, as compared to floxed controls ( Figure 6a–f; black outline). Conversely, accompanying the decrease in *Il10* expression in IL-12+IL-27 and/or IL-27-driven cultures, decreased expression of *Lars2, Id2, Hlx, Timp1, Pdpn* and *Rorc*, was observed in *Prdm1* ^fl/fl^ *Cd4* ^Cre^, *Maf* ^fl/fl^ *Cd4* ^Cre^ and *Prdm1* ^fl/fl^ *Maf* ^fl/fl^ *Cd4* ^Cre^ T cells, as compared to floxed controls, but *Lars2* and *Id2* were reduced to a lesser degree in *Maf* ^fl/fl^ *Cd4* ^Cre^ T cells in IL-12+IL-27-driven cultures ( Figure 5j–o; Supplementary Tables 5 and 6 ^30^), suggesting that Blimp-1 and c-Maf regulate common and distinct genes/pathways to enforce a regulated immune response. These data support the alternative *k-*means clustering analysis approach ( Figure 5j–o) described above. 

### *Prdm1* and *Maf* have complementary roles in regulating *Il10* and *Ifng* expression in IL-12, IL-12+IL-27 and IL-27-driven T cells

**Figure 7.**
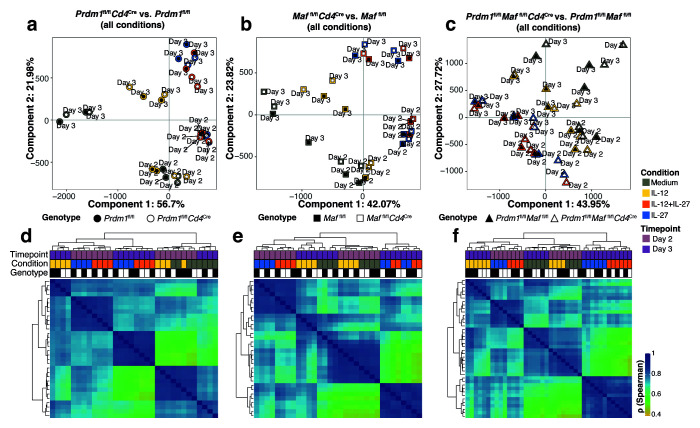
ATAC-seq reveals chromatin remodelling occurring between Day 2 and Day 3 in *in vitro* differentiated CD4 ^+^ T cells in the presence of IL-27, but no major changes in accessibility upon *Cd4* ^Cre^-mediated deletion of *Prdm1*, *Maf,* or both *Prdm1* and *Maf*. ATAC-seq analysis of CD4 ^+^ T cells differentiated *in vitro* in the presence of Medium, IL-12, IL-12+IL-27, or IL-27 on Day 2 and Day 3 with *Cd4* ^Cre^-mediated deletion of either *Prdm1*, *Maf*, or both *Prdm1* and *Maf,* and corresponding floxed controls. **a**– **c**, PCA plots showing PC1, explaining cell differentiation in the presence of IL-27, versus PC2, explaining transition from Day 2 to Day 3. **d**– **f**, Unsupervised hierarchical clustering of a pair-wise Spearman correlation of read coverages underlying ATAC-seq peaks called in *in vitro* differentiated CD4+ T cells in the presence of Medium, IL-12, IL-12+IL-27, or IL-27 from Day 2 to Day 3.

To identify the molecular mechanisms whereby Blimp-1 and c-Maf affected gene regulation in *in vitro* differentiated CD4 ^+^ T cells from the different CD4-specific transcription factor deleted mice, we used the assay for transposase-accessible chromatin plus sequencing (ATAC-seq) to reveal changes in functionally active genomic regions across each cytokine-driven condition. Consistent with the RNA-seq profile ( Figure 5a–c), ATAC-seq revealed that the cytokines added during culture was the most dominant variable shaping the open chromatin landscape of *in vitro* differentiated CD4 ^+^ T cells ( Figure 7a–c). Specifically, the principal component 1 (explaining 42-56% of the variance) segregated *in vitro* differentiated CD4 ^+^ T cells in the presence of IL-12 or medium from those differentiated in the presence of IL-12+IL-27 or IL-27. Moreover, major chromatin remodelling occurring between Day 2 and Day 3 was observed in all *in vitro* differentiated CD4 ^+^ T cells in the presence of cytokines, but no major changes in accessibility were observed upon *Cd4* ^Cre^-mediated deletion of *Prdm1*, *Maf,* or both *Prdm1* and *Maf* ( Figure 7a–f). Together these results highlight a role for IL-27 in influencing the open chromatin landscape of *in vitro* differentiated CD4 ^+^ T cells and suggests that c-Maf and Blimp-1 do not drive chromatin remodelling in order to perform their gene regulation functions, as has been shown for c-Maf ^20^.

**Figure 8.**
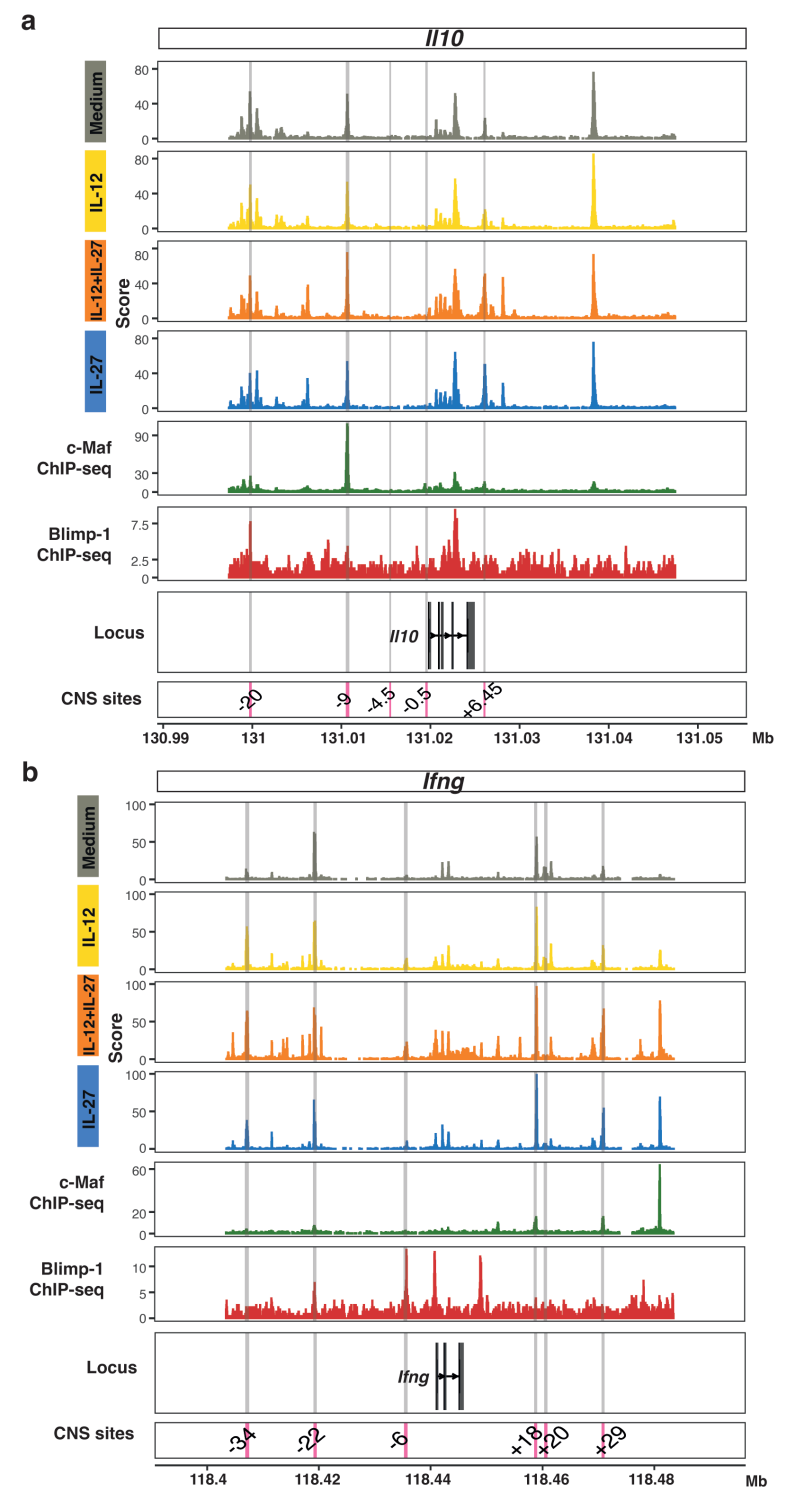
Integration of multiomic datasets identifies *Il10* and *Ifng* as targets for reciprocal regulation by Blimp-1 and c-Maf. Genome browser tracks of ATAC-seq data for **a**, *Il10* and **b**, *Ifng* from Day 3 CD4 ^+^ T cells differentiated in the presence of Medium, IL-12, IL-12+IL-27, and IL-27. ChIP-seq tracks for c-Maf (green, GSE40918) and Blimp-1 (red, GSE79339) are shown. CNS reported in the literature for *Il10* and *Ifng* are highlighted in grey shading and labelled in the bottom track. Data from n=2–3 biological replicates.

 Analysis of public c-Maf and Blimp-1 ChIP-seq datasets against our ATAC-seq data from T cells differentiated with IL-27+IL-12, IL-27 or IL-12, as compared to medium control, confirmed binding of Blimp-1 and c-Maf at accessible chromatin regions within the *Il10* locus ( Figure 8a). These findings further support the hypothesis that Blimp-1 and c-Maf may be critical regulators of IL-10 in multiple settings including Th1 cells differentiated with IL-12+IL-27 in addition to in “Tr1” cells differentiated with IL-27 as reported ^13^. We additionally show here that both transcription factors also bind accessible chromatin within the *Ifng* locus ( Figure 8b). Thus, binding of both Blimp-1 and c-Maf to the *Il10* and *Ifng* loci indicate that Blimp-1 and c-Maf are direct positive regulators of *Il10,* and direct negative regulators of *Ifng* ( Figure 8a and b). Blimp-1 and c-Maf showed binding to the *Il10* and *Ifng* locus at distinct sites, which suggests complementary action to induce *Il10* while reducing *Ifng* expression ( Figure 8a and b). Distinct c-Maf and Blimp-1 *Ifng* binding sites were also observed by ATAC-seq analysis and these increased in T cells differentiated with IL-12 and IL-12+IL-27 and to a lesser extent in IL-27-driven T cells ( Figure 8b).

### Gene regulatory networks derived from multiomic data integration highlight shared and unique targets of Blimp-1 and c-Maf

**Figure 9.**
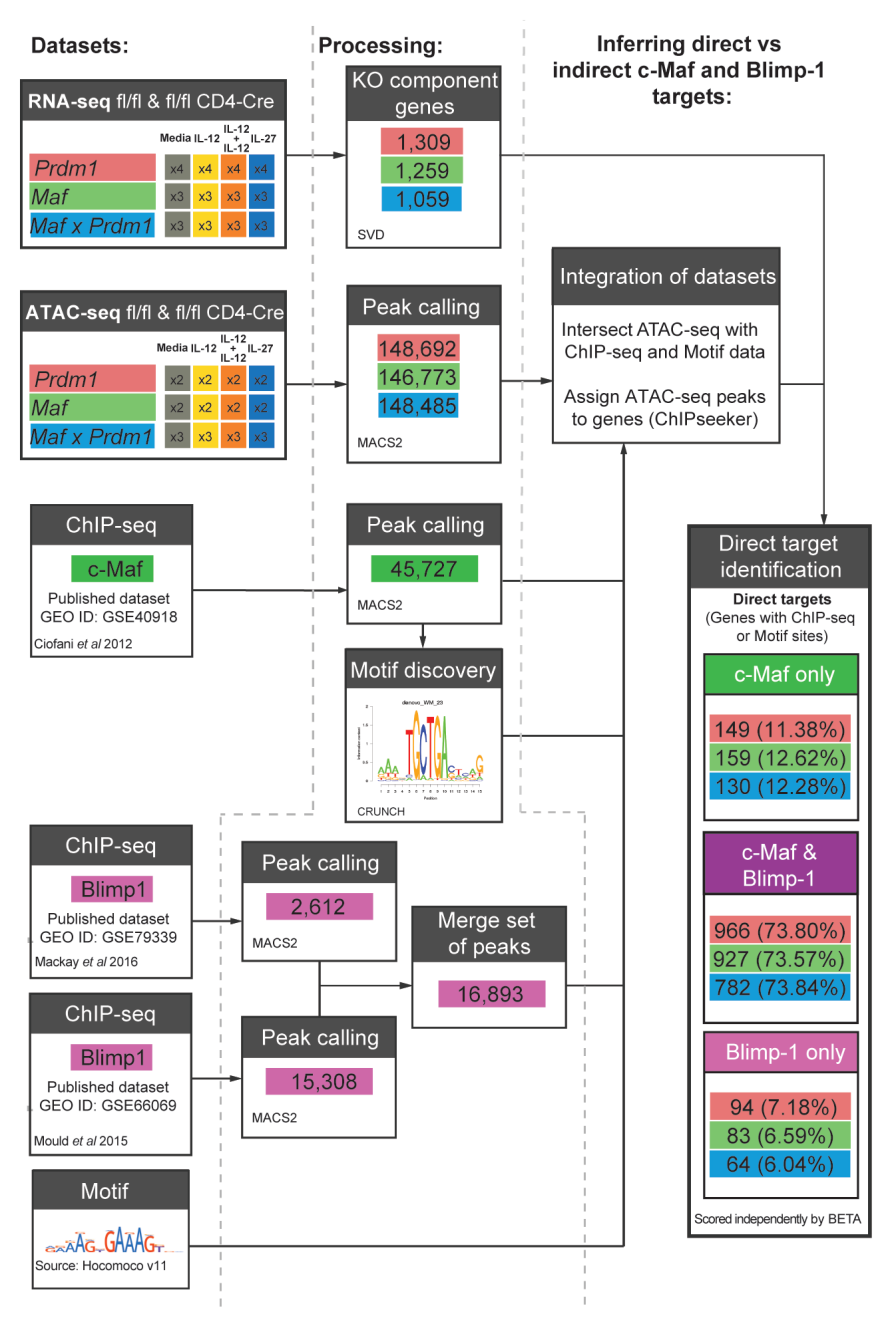
Schematic of framework applied to integrate multiomic datasets. Framework schematic for the identification of putative direct target genes of Blimp-1 and c-Maf in CD4+ T cells differentiated *in vitro* in the presence of Medium, IL-12, IL-12+IL-27, or IL-27 on Day 3, with replicates indicated. For each condition, Blimp-1 (GSE79339 and GSE66069) and c-Maf (GSE40918) ChIP-seq peaks and their corresponding motifs were filtered with and associated to the ATAC-seq peaks to identify the biologically relevant binding sites of Blimp-1 and c-Maf. Each ATAC-seq peak was associated to a gene based on distance proximity, thus, allowing the association of changes in the transcriptome with the binding of Blimp-1 and/or c-Maf (see Methods). Data from n=2–4 biological replicates.

**Figure 10.**
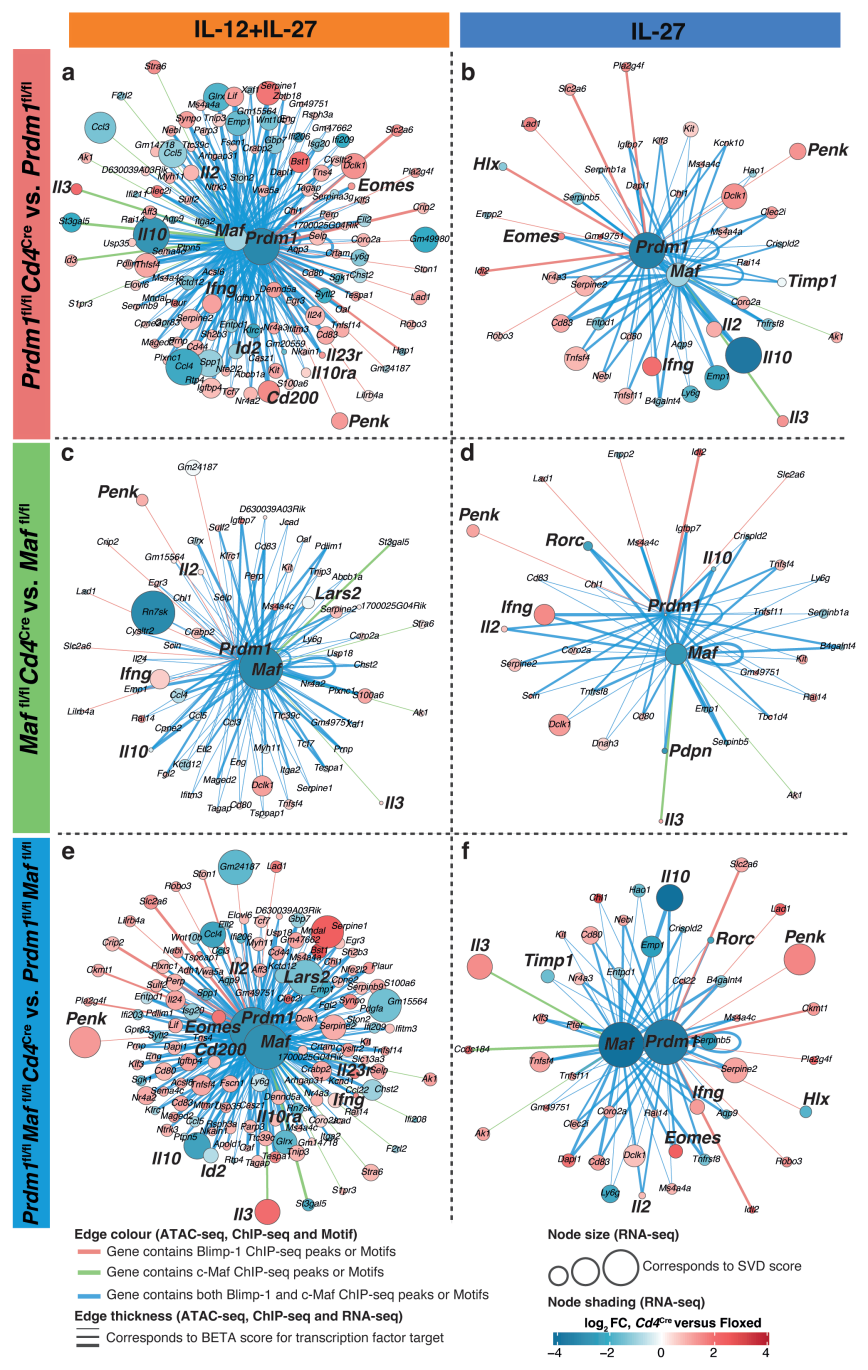
Gene regulatory networks derived from multiomic data integration highlight a majority of shared targets between Blimp-1 and c-Maf affected by *Cd4* ^Cre^-mediated deletion of *Prdm1*, *Maf*, or both *Prdm1* and *Maf*. RNA-seq, ATAC-seq, ChIP-seq, and motifs were integrated (see Methods and Figure 9) to derive gene regulatory networks of the direct targets of Blimp-1 and/or c-Maf. Direct targets of Blimp-1 and/or c-Maf that were most affected upon *Cd4* ^Cre^-mediated deletion of **a**, *Prdm1*, **b**, *Maf*, or **c**, both *Prdm1* and *Maf* with IL-12+IL-27 (Clusters 2,1,7 from Figure 5j) and IL-27 alone (Clusters 5, 7, 1 from Figure 5m) differentiated CD4+ T cells *in vitro*. In the networks, the nodes correspond to genes affected upon *Cd4* ^Cre^-mediated deletion of the transcription factors. The node size reflects the contribution of a gene to the SVD component associated with the deletion of the transcription factors. The node colouring represents the fold change of the *Cd4* ^Cre^-mediated knockouts compared to the floxed control gene expression. The edge colour depicts if Blimp-1 (pink), c-Maf (green), or both Blimp-1 and c-Maf (blue) have binding sites assigned to the target genes; whilst the thickness of the edge shows the likelihood of c-Maf and/or Blimp-1 regulating a gene according to the BETA software. Data incorporates the RNA-seq and ATAC-seq biological replicates outlined in Figure 9 schematic. Data from n=2–4 biological replicates.

We further integrated our RNA-seq data and ATAC-seq data with data obtained by analysis of public Blimp-1 and c-Maf ChIP-seq datasets ^20, 49^ and public motif data to identify genes that were targets of Blimp-1 and c-Maf ( Figure 9 schematic). These results were confirmed by BETA software, which integrates ChIP-seq analyses and gene-expression data to identify target genes ( Figure 9). Gene regulatory networks derived from the multiomic data integration highlighted unique and shared targets between Blimp-1 and c-Maf affected by *Cd4* ^Cre^-mediated deletion of *Prdm1, Maf* or both *Prdm1* and *Maf* ( Figure 9). Direct targets of Blimp-1 and/or c-Maf that were the most affected upon *Cd4* ^Cre^-mediated deletion of *Prdm1*, *Maf*, or both *Prdm1* and *Maf* with IL-12+IL-27 were found in Clusters 2,1,7 from Figure 5j and for IL-27 alone in Clusters 5, 7, 1 from Figure 5m, and are depicted as gene regulatory networks in Figure 10a, 10c and 10e and Figure 10b, 10d and 10f respectively. In the networks shown in Figure 10a, 10c and 10e (IL-12+IL-27) and in Figure 10b, 10d and 10e (IL-27 alone), the nodes correspond to genes affected upon *Cd4* ^Cre^-mediated deletion of the transcription factors, with the node size reflecting the contribution of a gene to the SVD component associated with the deletion of the transcription factors; the node colouring represents the fold change of the *Cd4* ^Cre^-mediated knockouts compared to the floxed control gene expression (red up, blue down-regulated). The edge colour depicts if Blimp-1 (pink), c-Maf (green), or both Blimp-1 and c-Maf (blue) have binding sites assigned to the target genes; whilst the thickness of the edge shows the likelihood of c-Maf and/or Blimp-1 regulating a gene according to the BETA software. Data incorporates the RNA-seq and ATAC-seq biological replicates as outlined in the Figure 9 schematic.

In IL-12+IL-27-differentiated Th1 cells, shared targets included *Il10, Id2, Ccl3, Ccl4, Ccl5,* which were reduced upon *Cd4* ^Cre^-mediated deletion of *Prdm1*, both *Prdm1* and *Maf* and to a lesser extent *Maf,* as compared to floxed controls ( Figure 10a, 10c and 10e; Supplementary Table 7 ^30^). Both *Prdm1* and *Maf* were shown to be direct targets of each other and were reduced in the absence of the reciprocal transcription factor ( Figure 10a, 10c and 10e; Supplementary Table 7 ^30^). Blimp-1 and/or c-Maf targets of genes which were upregulated in Th1 cells differentiated with IL-12+IL-27 upon *Cd4* ^Cre^-mediated deletion of both *Prdm1* and *Maf,* but to a lesser extent upon *Prdm1*, and to a far lesser degree upon *Maf* deletion, as compared to floxed controls, included *Ifng, Il2, Il3, Tcf7, Tnfsf4, Cd80, Cd83, Eomes, Serpine1/2, Penk, Cd200, Il23r* ( Figure 10a, 10c and 10e; Supplementary Table 7 ^30^). Under these differentiation conditions unique direct targets of c-Maf included *Il3* and *Stra6 (*encoding the Vitamin A Receptor) and of Blimp-1 included *Penk,* and these genes were the most upregulated in the Th1 cells upon *Cd4* ^Cre^-mediated deletion of either *Prdm1* or both *Prdm1* and *Maf,* and to a lesser extent upon *Maf* deletion, as compared to floxed controls ( Figure 10a, 10c and 10e; Supplementary Table 7 ^30^). In T cells differentiated with IL-27 alone, shared targets again included *Il10,* but also *Timp1, Pdpn, Rorc,* and these were reduced upon *Cd4* ^Cre^-mediated deletion of both *Prdm1* and *Maf* and either *Prdm1* or *Maf* alone, as compared to floxed controls ( Figure 10b, 10d and 10f). Shared targets of Blimp-1 and c-Maf which were upregulated in IL-27 differentiated T cells upon *Cd4* ^Cre^-mediated deletion of either *Prdm1* or both *Prdm1* and *Maf,* and to a much lesser extent in *Maf,* as compared to floxed controls included *Il2, Ifng, Tnfsf4* and *Eomes* ( Figure 10b, 10d and 10f; Supplementary Table 7 ^30^). Unique targets of Blimp-1 were revealed in IL-27 differentiated T cells and included *Hlx,* which was reduced and *Penk* which was increased upon *Cd4* ^Cre^-mediated deletion of *Prdm1,* as compared to floxed controls ( Figure 10b, 10d and 10f; Supplementary Table 7 ^30^). Unique targets of c-Maf included *ll3* which was increased upon *Cd4* ^Cre^-mediated deletion of both *Prdm1* and *Maf* and either *Prdm1* or *Maf* alone ( Figure 10b, 10d and 10f; Supplementary Table 7 ^30^). Under IL-27 differentiation conditions both *Prdm1* and *Maf* were also shown to be direct targets of each other and were reduced in the absence of the reciprocal transcription factor ( Figure 10b, 10d and 10f; Supplementary Table 7 ^30^).

**Figure 11.**
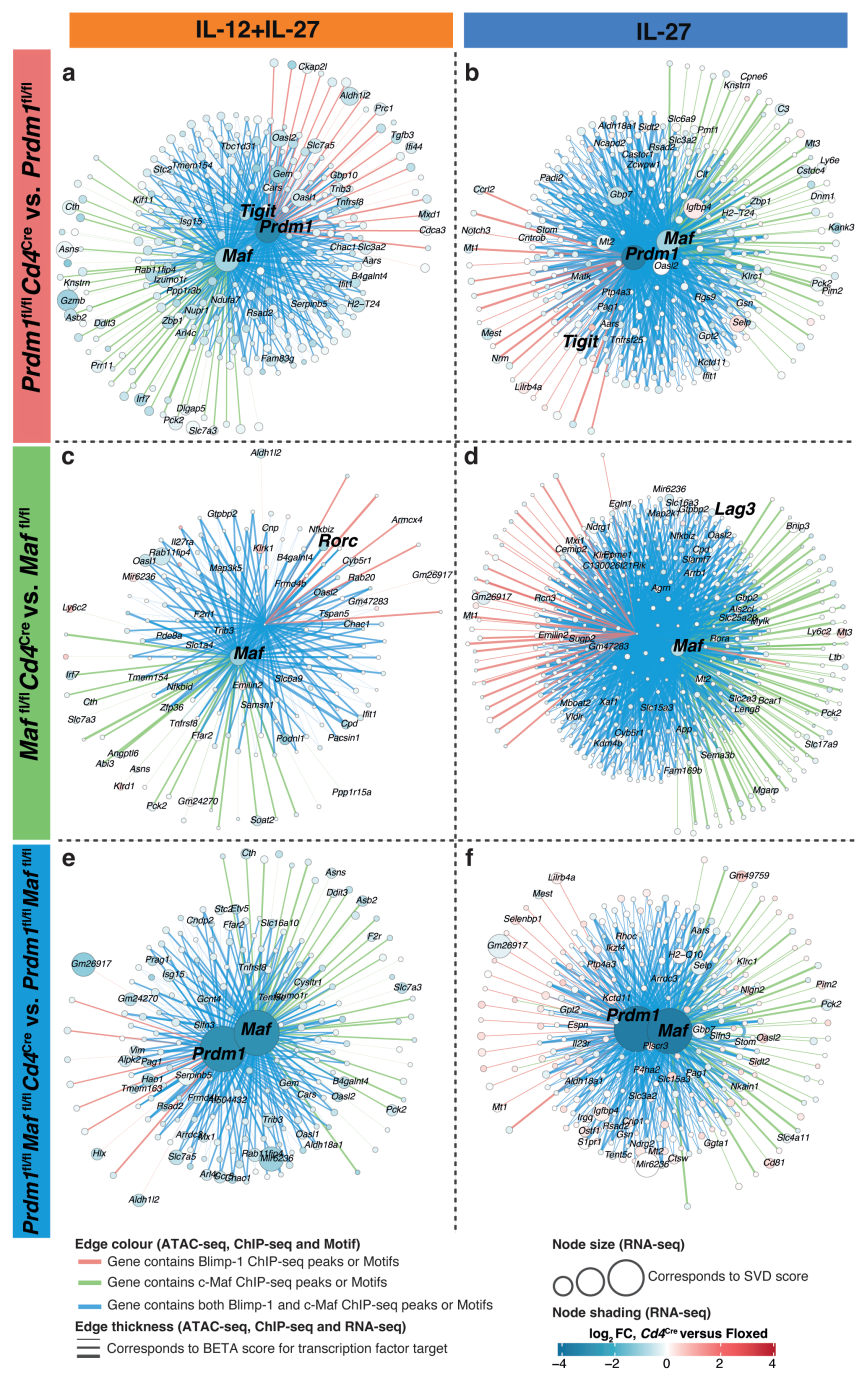
Gene regulatory networks derived from multiomic data integration point to known and new shared targets between Blimp-1 and c-Maf. Gene regulatory networks of the direct targets of Blimp-1 and/or c-Maf were derived, as in Figure 10, for clusters that contained genes that had been previously been reported to be regulated by c-Maf and Blimp-1 and were affected upon *Cd4* ^Cre^-mediated deletion of **a**, *Prdm1*, **b**, *Maf*, or **c**, both *Prdm1* and *Maf* with IL-12+IL-27 (Clusters 5 from Figure 5j) and IL-27 alone (Clusters 6 from Figure 5m) differentiated CD4+ T cells *in vitro*.

Additionally, further network analysis of the IL-12+IL-27 CD4 T differentiated cell data ( Figure 11a, 11c and 11e; Supplementary Table 7 ^30^) was applied to “Cluster 5” (from Figure 5j) with addition of the *Prdm1* and *Maf* genes and to IL-27 CD4 T differentiated cell data ( Figure 11b, 11d and 11f; Supplementary Table 7 ^30^) to “Cluster 6” of Figure 5m again with addition of the *Prdm1* and *Maf* genes. Although weakly affected target genes were observed in these clusters that were affected by *Cd4* ^Cre^-mediated deletion of *Prdm1*, both *Prdm1* and *Maf* as compared to floxed controls, these clusters included *Tigit, Lag3* in keeping with previous reports ^13, 64^.

**Figure 12.**
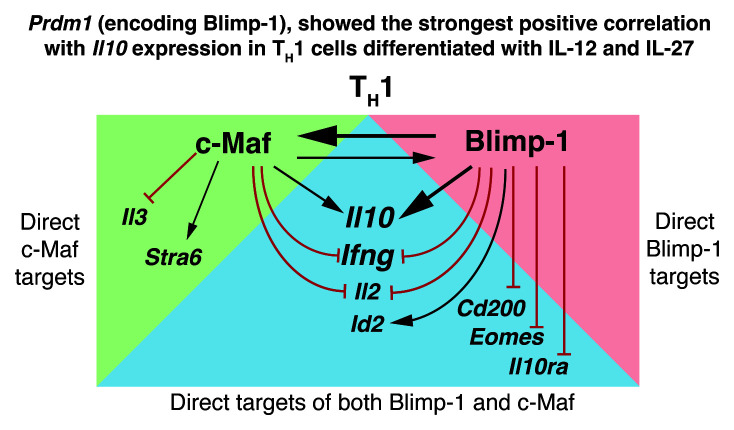
Comprehensive transcriptomic analysis reveals that Blimp-1 and c-Maf regulate *Il10*, cross-regulate each other, but also negatively regulate common and unique proinflammatory gene networks. Summary schematic of some of the findings herein presented in regards of the genes regulated by c-Maf and Blimp-1. The vast majority of direct targets detected were shared between c-Maf and Blimp-1, however they were affected to different extents upon the *Cd4* ^Cre^-mediated deletion of *Prdm1*, *Maf*, or both *Prdm1 and Maf*.

In summary we show that c-Maf and Blimp-1 are direct targets of each other, which regulate each other to directly induce *Il10* gene expression in Th1 cells differentiated in IL-12+IL-27 ( Figure 12). We show that *Il10* is a direct target of both transcription factors in Th1 cells as previously reported for ‘Tr1 cells’ differentiated with IL-27 alone ^13^. We additionally show that both c-Maf and Blimp-1 can directly bind to the proinflammatory cytokine loci *Ifng, Il2* and *Id2* in IL-12+IL-27 differentiated Th1 cells and negatively regulate their expression, thus enforcing a controlled Th1 effector response. Moreover, c-Maf binds and positively regulates *Stra6* and binds and negatively regulates *Il3;* while Blimp-1 binds and positively regulates *Id2* and binds and negatively regulates *Cd200* and *Eomes* ( Figure 12). Importantly, these genes are most strongly affected by *Cd4* ^Cre^-mediated deletion of both *Prdm1* and *Maf.*

Our study shows that the transcription factors Blimp-1 and c-Maf are co-dominant positive regulators of *Il10* in IL-12+IL-27-driven Th1 cells as was recently reported for IL-27-driven “Tr1” cells ^13^. We additionally show that both Blimp-1 and c-Maf also negatively co-regulate common and unique proinflammatory gene networks in both IL-12 plus IL-27 differentiated Th1 cells and IL-27-differentiated “Tr1”cells. These data demonstrate that together Blimp-1 and c-Maf control a network of genes to ensure a tightly regulated IL-12-driven Th1 effector responses to limit host damage.

## Discussion

Using computational inference of gene regulation derived from temporal gene cluster profiling and analysis of active genomic regions in Th1 cells differentiated with IL-12 and IL-27 or IL-12 alone, which produce proinflammatory cytokines but differ with respect to *Il10* expression, we show that Blimp-1 and c-Maf are co-dominant transcriptional regulators of *Il10* gene expression. We confirm these findings using T-cell specific deletion of these transcription factors and show that both transcription factors additionally negatively regulate a network of proinflammatory effector genes in Th1 cells through their indirect and direct action on shared and distinct effector target genes, thus reinforcing a controlled Th1 cell response.

Temporal profiling of gene expression has been reported to facilitate the development of regulatory transcriptional networks dictating the differentiation of naïve CD4 ^+^ T cells in Th17 cells ^20, 65^, and Th2 cells ^66^, leading to the discovery of key regulators of activation and differentiation and reinforcing the general principles for T helper cell differentiation. We herein applied clustering algorithms and temporal profiling to gene expression data together with analysis of active genomic regions to reveal the transcriptional networks regulating *Il10* expression against that of the Th1-specific proinflammatory cytokine *Ifng*. Data from these analyses were compared across Th1 cells differentiated with IL-12 plus IL-27, which expressed high levels of both *Ifng* and *Il10*, and Th1 cells differentiated with IL-12 alone, which expressed high levels of *Ifng* but no *Il10*, alongside T cells differentiated with IL-27 alone, often referred to as ‘Tr1 cells’ ^13^, which expressed *Il10* but little to no *Ifng* and control CD4 ^+^ T cells cultured in medium alone where *Il10* or effector cytokine expression is below the level of detection. This comparative analysis of *Il10* expression against *Ifng* and other effector cytokines allowed us to identify transcription factors predicted to be positive regulators of *Il10*. Additionally, this computational analysis allowed us not only to identify putative co-dominant transcription factors regulating *Il10* but additionally to determine any effects on *Ifng* and effector cytokine gene expression and transcription factors associated with Th1 cell differentiation. Th1 cells producing IFN-γ and IL-10 have been shown to be required for control of Th1 cell responses *in vivo* during chronic infection with intracellular pathogens to inhibit collateral host damage ^10, 11, 14, 15^, whereas Th1 cells producing only IFN-γ have been associated with acute infection ^67, 68^. CD4 ^+^ T cells producing IL-10 only in the absence of proinflammatory cytokines have been described ^69, 70^ and often referred to as ‘Tr1 cells’ ^13, 71^, which *in vivo* have been reported in the intestine ^69, 72, 73^ although it is unclear whether they are effector Th cells which have diminished their proinflammatory cytokine expression whilst maintaining IL-10 production in the intestine ^72^ or under certain metabolic conditions ^74^. Therefore, a detailed knowledge of transcriptional regulation of *Il10* expression and effector cytokines such as *Ifng* and accompanying Th1 effector cytokines may provide therapeutic avenues to target inflammatory disease.

Analysis of transcription factors revealed that *Prdm1* showed the most significant positive correlation with *Il10* expression followed by *Id2, Asb2, Hlx, Nfatc2* and *Maf,* some of which we and others have previously reported as regulators of *Il10* ^9, 13, 18, 19, 68^. Other proposed transcription factors reported to regulate *Il10* expression in T cells, such as *Hif1a* and *Nfil3* ^9, 13, 19, 68^ showed only a slight correlation with *Il10* expression in Th1 cells differentiated with IL-12 plus IL-27 and were also expressed in Th1 cells differentiated with IL-12 alone, which did not express *Il10,* suggesting that they are not directly involved in *Il10* gene expression. Likewise, the transcription factor *Batf,* which has been previously reported to regulate *Il10* expression in differentiating Th2 cells ^75^ was actually down-regulated in both IL-12 plus IL-27 and IL-27 alone differentiation conditions, whilst increasing under IL-12 alone differentiation conditions, and showed very poor correlation with *Il10* expression. Thus, as discussed earlier for IL-21, it is likely that a number of these transcription factors are required for the differentiation and possibly proliferation of T helper cells rather than for direct positive regulation of *Il10* gene expression.

Analysis of transcriptional activity from ATAC-seq data, using BaGFoot software ^39^ revealed differential transcriptional activity for the transcription factors *Prdm1* and *Maf* between day 2 and day 3 in IL-12 plus IL-27 differentiated Th1 cells and IL-27 differentiated T cells, which both express *Il10.* These transcription factors were not found to be significantly active between day 2 and day 3 in IL-12 differentiated Th1 cells or medium control cultured CD4 ^+^ T cells, which do not express *Il10,* suggesting that these transcription factors may be co-dominant regulators of *Il10.* By contrast, the transcriptional activity of the AP-1 family member, *Batf,* was only evident in cells cultured in medium alone or IL-12, which do not express *Il10*. Other AP-1 family members *Jun* and *Fos* showed increased transcriptional activity across all conditions including in IL-12-driven Th1 cells producing *Ifng* and no *Il10,* as well as in *Il10* expressing cells. This suggests that Batf and other AP-1 family members may be pioneer factors involved in Th cell differentiation, as previously suggested ^76^, rather than major regulators of *Il10* ^75^ and endorses the role of Jun and Fos as enhancers of *Il10* gene regulation ^6, 57^. Increased transcriptional activity of *Stat 3, 4* and *5* was most pronounced under IL-12 conditions in Th1 cells expressing *Ifng* but not *Il10.* Transcriptional activity of *Bhlhe40*, a known negative regulator of IL-10 ^16^ and which we have previously shown to be negatively regulated by c-Maf, was found to be increased in cells cultured in IL-12, IL-12+IL-27 and IL-27, supporting its role as a regulator of *Il10* ^16^. Our findings suggesting that *Prdm1* and *Maf* are co-dominant regulators of *Il10* in IL-12 plus IL-27 differentiated Th1 effector cells are in keeping with the report from Kuchroo *et al.* ^13^, who recently computed a transcriptional network induced by IL-27 in CD4 ^+^ T cells, termed ‘Tr1 cells’, expressing *Il10,* but little to no *Ifng.* Hence *Prdm1* and *Maf* not only promote *Il10* expression in a T cell regulatory setting such as ‘Tr1 cells’ as reported, but additionally appear to be co-dominant regulators of *Il10* in an effector Th1 setting accompanying high levels of *Ifng.* Indeed, specific deletion of *Prdm1, Maf* and the combination of both these transcription factors in IL-12 plus IL-27 differentiated Th1 effector cells expressing *Ifng,* confirmed their co-dominant role in regulating *Il10* gene expression in these pro-inflammatory cells. Thus, *Prdm1* and *Maf* are not only central hubs in regulating the expression of *Il10* in IL-27 differentiated ‘Tr1 cells’ where they were confirmed to control a regulatory circuit of multiple other transcriptional modulators using Prdm1/Maf DKO ‘Tr1 cells’ ^13^, but as we now show, also regulate *Il10* expression in a proinflammatory Th1 effector setting.

Commitment of T helper cells to specific subsets requires induction of master transcription factors that induce specific transcriptional programs that direct a specific T cell subset towards terminal differentiation while restricting the fates of other T cell subsets ^77^. Thus, we questioned whether *Prdm1* and *Maf* may be part of the network for Th1 cell differentiation with *Il10* expression accompanying terminal differentiation of these cells to provide feedback regulation, or alternatively contribute to a regulated Th1 response by antagonising the expression of *Ifng* and other proinflammatory molecules. The absence of *Prdm1* and *Maf* in IL-12 plus IL-27 differentiated Th1 cells actually resulted in an increase in *Ifng* expression, showing that while *Prdm1* and *Maf* synergistically promote *Il10* expression, they negatively regulate the expression of the effector cell programme, reflected by increased expression of *Ifng,* thus controlling Th1 effector responses. It is likely that this co-dominant transcriptional regulation by *Prdm1* and *Maf* is in place to ensure a controlled Th1 response against chronic infection with intracellular pathogens to minimise accompanying pathology. The negative regulation of *Ifng* that we observed was most pronounced in Th1 cells differentiated in IL-12 plus IL-27. This was in contrast to the discussion from Kuchroo *et al.* ^13^, in IL-27-only driven IL-10 producing ‘Tr1 cells’, which expressed minimal to no *Ifng,* that although *Prdm1* and *Maf* synergistically promoted IL-10 production, they did not inhibit production of T helper cell signature cytokines. This may reflect the differential effects on distinct T cell subsets. However, our findings using in-depth clustering of RNA-seq data demonstrated that T-cell specific deletion of *Prdm1*, *Maf*, or both transcription factors, led to an increase in several proinflammatory genes in both IL-27 differentiated T cells as well as IL-12 plus IL-27 differentiated Th1 cells, although to a much larger extent (expression and number of genes) in the Th1 cells. Although we found that the absence of *Prdm1, Maf* or both transcription factors resulted in an increase in *Ifng* expression, the expression of the Th1/IFN-specific transcription factor *Tbx21* ^63^ was not significantly affected, suggesting that Blimp-1 and c-Maf may potentially have direct effects on the *Ifng* gene itself. This was supported by combining ATAC-seq and ChiP-seq data, which clearly revealed both unique and overlapping Blimp-1 and c-Maf sites not only in the *Il10* locus, but additionally in the *Ifng* locus. Moreover, Th1 cells differentiated in IL-12 (together with IL-27 or not) resulted in increased chromatin accessibility in the *Ifng* locus possibly enhancing the regulatory action of *Prdm1* and *Maf.*

Additionally, gene regulatory networks derived from multiomic data integration highlighted a large number of shared and some unique targets of Blimp-1 and c-Maf which showed positive and negative regulatory effects on gene expression. Accompanying these dominant effects upon deletion of *Prdm1* and *Maf* on *Il10* expression in IL-12 plus IL-27 driven Th1 cells, was a decrease in other genes including *Id2, Lars2* and *Tigit,* whilst in IL-27 driven T cells the decrease in *Il10* was accompanied by a decrease in expression of genes including *Timp1, Hlx, Tigit*, *Rorc,* in keeping with the reports from Kuchroo on *Il10*-only producing ‘Tr1 cells’ ^13^, while deletion of both *Prdm1* and *Maf* in IL-12 plus IL-27 differentiated Th1 cells led to an increased number as well as level of proinflammatory gene expression including, *Ifng, Il23r, Eomes, Il2,Il3, Penk* and *Cd200* and others, this was mostly less marked in the IL-27 alone differentiated cells, although many were found to be shared targets of both *Prdm1* and *Maf.*

In summary, we have shown that *Prdm1* and *Maf* are co-dominant transcription factors that induce *Il10* gene expression, together with a cluster of genes including other transcription factors and co-inhibitory receptor genes, indicating their role in establishing an immunoregulatory gene programme in T cells. In addition, our findings show that both *Prdm1* and *Maf* also negatively regulate a number of proinflammatory genes including *Ifng, Il23r, Eomes, Il2, Il3, Penk* and *Cd200* and others, most strongly in Th1 cells, demonstrating their major role in controlling Th1 responses to allow eradication of pathogens with minimum pathology.

## Data Availability

The materials, data and any associated protocols that support the findings of this study are available from the corresponding author upon request. The RNA-seq and ATAC-seq datasets have been deposited in the NCBI Gene Expression Omnibus (GEO) database with the primary accession number GSE197789. Publicly available datasets used in this study include GSE40918, GSE79339, and GSE66069. GEO: Blimp-1 and c-Maf regulate Il10 and negatively regulate common and unique proinflammatory gene networks in IL-12 plus IL-27-driven T helper-1 cells [Mus musculus (house mouse)]. Accession number GSE197789; https://identifiers.org/geo:GSE197789 ^78^ GEO: A validated regulatory network for Th17 cell specification [Mus musculus (house mouse)]. Accession number GSE40918; https://identifiers.org/geo:GSE40918 ^20^ GEO: Hobit and Blimp1 instruct a universal transcriptional program of tissue-residency in lymphocytes [Mus musculus (house mouse)]. Accession number GSE79339; https://identifiers.org/geo:GSE79339 ^49^ GEO: Analysis of Blimp-1 and Irf-1 genomic binding in wild type and Prdm1/Blimp-1 mutant embryonic gut [Mus musculus (house mouse)]. Accession number GSE66069; https://identifiers.org/geo:GSE66069 ^50^ Figshare: Blimp-1 and c-Maf regulate *Il10* and negatively regulate common and unique proinflammatory gene networks in IL-12 plus IL-27-driven T helper-1 cells. https://doi.org/10.6084/m9.figshare.23592393 ^30^ This project contains the following underlying data: SupplementaryTable1_KineticsRawGeneCounts.xlsx SupplementaryTable2_KineticsDifferentialGeneExpression_KmeansCluster.xlsx SupplementaryTable3_TF_Cytokines_GeneLists.xlsx SupplementaryTable4_CD4creDeletion_FloxCtrls_RawNormGeneCounts SupplementaryTable5_SVDresults.xlsx SupplementaryTable6_SVD_CD4CreDeletionComponent_KmeansClusterFoldChanges.xlsx SupplementaryTable7_DirectTargets_Blimp1-cMaf_NetworkValues.xlsx Data are available under the terms of the Creative Commons Attribution 4.0 International license (CC-BY 4.0). Repository: ARRIVE checklist for ‘Blimp-1 and c-Maf regulate *Il10 and* negatively regulate common and unique proinflammatory gene networks in IL-12 plus IL-27-driven T helper-1 cells’. https://doi.org/10.6084/m9.figshare.23592393 ^30^ Data are available under the terms of the Creative Commons Attribution 4.0 International license (CC-BY 4.0).
