# Supplementary material for: Blimp-1 and c-Maf regulate  Il10 and negatively regulate common and unique proinflammatory gene networks in IL-12 plus IL-27-driven T helper-1 cells
Source: Wellcome Open Res. 2023 Dec 1;8:403. Originally published 2023 Sep 18. [Version 2] doi: 10.12688/wellcomeopenres.19680.2 (PMC10709690; doi:10.12688/wellcomeopenres.19680.2)
Supplement: Peer review history

# Reviewer report: Reviewer response for version 2

**DOI:** 10.21956/wellcomeopenres.22787.r70937

**Published:** 2023-12-08

**Authors:** Leonie Taams

**Affiliations:** 1 King's College London, London, England, UK

I would like to thank the authors for their detailed and comprehensive replies and edits; I am happy to state that I am satisfied with these revisions.

Is the work clearly and accurately presented and does it cite the current literature?

Partly

If applicable, is the statistical analysis and its interpretation appropriate?

I cannot comment. A qualified statistician is required.

Are all the source data underlying the results available to ensure full reproducibility?

Yes

Is the study design appropriate and is the work technically sound?

Yes

Are the conclusions drawn adequately supported by the results?

Yes

Are sufficient details of methods and analysis provided to allow replication by others?

Yes

Reviewer Expertise:

Immunoregulation (wet-lab based) with limited expertise in bioinformatic analysis

I confirm that I have read this submission and believe that I have an appropriate level of expertise to confirm that it is of an acceptable scientific standard.

# Reviewer report: Reviewer response for version 2

**DOI:** 10.21956/wellcomeopenres.22787.r70938

**Published:** 2023-12-08

**Authors:** Christopher A Hunter

**Affiliations:** 1 Department of Pathobiology, University of Pennsylvania, Philadelphia, PA, USA

This is good to go. In particular the explanation for why all the data sets are included (as part of a resources paper) makes this clearer to this reviewer.

Is the work clearly and accurately presented and does it cite the current literature?

Yes

If applicable, is the statistical analysis and its interpretation appropriate?

Yes

Are all the source data underlying the results available to ensure full reproducibility?

Yes

Is the study design appropriate and is the work technically sound?

Yes

Are the conclusions drawn adequately supported by the results?

Yes

Are sufficient details of methods and analysis provided to allow replication by others?

Partly

Reviewer Expertise:

NA

I confirm that I have read this submission and believe that I have an appropriate level of expertise to confirm that it is of an acceptable scientific standard.

# Reviewer report: Reviewer response for version 1

**DOI:** 10.21956/wellcomeopenres.21798.r67170

**Published:** 2023-10-19

**Authors:** Christopher A Hunter

**Affiliations:** 1 Department of Pathobiology, University of Pennsylvania, Philadelphia, PA, USA

The overarching premise of this study is that transcriptionally distinct subsets of T lymphocytes are required for control and eradication of intracellular pathogens, however the transcriptional networks within specialized T cell subsets that regulate key cytokines required for control, including IFN-g and IL-10 remains unclear. Here they take a multi-modal approach to investigate the transcriptional networks underlying cytokine production within CD4 T cells treated in Th1 polarizing conditions in vitro. Utilizing various combinations of the cytokines IL-12 and IL-27. They generate and present original RNA and ATAC-seq datasets to elucidate the co-dominant role of *Prdm-1* (Blimp-1) and *Maf* (c-Maf) in regulating expression of IL-10 in Th1 cells, and this is reinforced and affirmed using mice with T-cell specific deletion of these transcription factors. This is similar (in some ways) to previous studies that used public and in-house transcriptional datasets to identify Prdm-1 and Maf as co-regulators of IL-10 production by Tr1 cells differentiated in vitro with IL-27.

 Our understanding of the molecular mechanisms that underly the integration of cytokine signals within a cell and drive functional cell states is continues to develop and this study provides a piece of the puzzle through their comprehensive analysis of the integration of signals for the IL-12 family members at the transcriptional and epigenetic level. The undertaking to generate and analysis high-dimensional datasets is no easy task and the breadth of the undertaking really makes this a nice resource data set. This iteration does distinguish itself from the previous report on Prdm-1 and Maf regulating IL-10 expression in Tr1 cells and its genuinely important to know what is robust and reproducible so there is clear value to this report. The authors do try to get the most out of their novel datasets, but in places the manuscript is dense and it might help to remove redundant figures and focus the narrative to drive home their points that Prdm-1 and Maf are regulators of IL-10 expression in a variety of conditions. However, as currently written the difference in transcriptional regulation between Tr1 and Th1 cells is unclear and the data sets need to be more fully synthesized. If I was reviewing this for a colleague – my suggestion would be to look again at the original sentence at the end of the first paragraph of the Introduction.  Maybe consider re-working the sentiment that the transcriptional mechanisms that regulate cytokine production by Th1 cells including their production of IL-10 are as yet unclear. It undersells what is = a rich literature on this topic that for example led to the identification of T-bet and how STATs promote Th1 responses and IL-10 production. Maybe this can be used to better rfame how the current study moves the field.

 The comments below are provided to help with clarity and are not meant to be prescriptive and new experiments or reanalysis is not requested.

 Figure 1 – no comments. Favorite figure.

 Figure 2 – These transcriptional data have already been presented within Figure 1, the only difference is the visualization used to display the data. The authors have already presented these genes and determined them to be DEGs in Figure 1C, and we are not sure how/if presenting the read counts provides any new insight. The authors already show a pearson’s correlation in Figure 1e and use the second half of Figure 2 to show correlations derived from a linear regression. It is unclear whether these graphs provide a novel lens to interpret these data. Perhaps Figure 1 is sufficient for the claims made in Figure 2?

 Figure 3 – the authors take advantage of a software package BaGFoot to analysis ATAC-seq data but there is not sufficient description of the package to allow a reader to interpret the graphs. The authors highlight that these depictions show increased transcriptional activity for Prdm1 and Maf, but does this provide additional insight beyond what is shown by figure 1. Perhaps just needs to be better integrated.

 Figure 4 – Good as is. We like the figure and the written description.

 Figure 5 – The authors move onto T-cell specific depletion of Prdm-1 and Maf and transcriptional analysis of these cells via RNA-seq. Is the variance (G-I) redundant with the graphs (D-F)?

 Figure 6 – all of these genes are already shown in Figure 5 and seems redundant.

 Figure 7 – The interpretation of the PCA analysis for this ATAC-seq data is similar to what has been previously established, although it is important to see the consistency. The authors go on to say that there is no chromatin remodeling, but there are differences in the visualizations of the heatmap. For example. it appears that the absence of Maf alone induces a similar chromatin remodeling to the absence of both Prdm1 and Maf. Are we interpreting this wrong?

 Figure 8 – Maybe there is a mis-understanding (on our part). How does the IL-10 and IFN-g locus look in the ATAC-seq data generated from T -cell specific depletion of Prdm-1 and Maf as shown in Figure 7. These data would be more interesting to see rather than the WT cells (described in the figure legend?).

 Fig 9, 10 and 11. The authors have done a lot of analysis but it would be genuinely helpful if they could provide clarity in the discussion as to why these genes are interesting. For example, the focus on Cluster 5 is unclear.

Is the work clearly and accurately presented and does it cite the current literature?

Yes

If applicable, is the statistical analysis and its interpretation appropriate?

Yes

Are all the source data underlying the results available to ensure full reproducibility?

Yes

Is the study design appropriate and is the work technically sound?

Yes

Are the conclusions drawn adequately supported by the results?

Yes

Are sufficient details of methods and analysis provided to allow replication by others?

Partly

Reviewer Expertise:

Cytokines and T cells

I confirm that I have read this submission and believe that I have an appropriate level of expertise to confirm that it is of an acceptable scientific standard, however I have significant reservations, as outlined above.

# Response

**Published:** 2023-11-23

**Authors:** Anne O'Garra

**Affiliations:** The Francis Crick Institute, UK

**Christopher A Hunter ** Department of Pathobiology, University of Pennsylvania, Philadelphia, PA, USA ***We thank this reviewer for his comments and suggestions which have helped to clarify apsects of our manuscript, however we would like to explain that our reason for providing all of these complementary analyses is in view of the fact this manuscript is submitted as a Resource paper and that Wellcome Open Research does not allow Supplementary Figures, or data not shown. Inclusion of all these complementary analyses will highlight these data which will be accessible for further analyses to specialist readers with further interest as the RNA-seq and ATAC-seq data will all be deposited in the GEO for public access. We have clarified our manuscript upon the suggestions of this reviewer and added or replaced text as we explain below.***

 The overarching premise of this study is that transcriptionally distinct subsets of T lymphocytes are required for control and eradication of intracellular pathogens, however the transcriptional networks within specialized T cell subsets that regulate key cytokines required for control, including IFN-g and IL-10 remains unclear. Here they take a multi-modal approach to investigate the transcriptional networks underlying cytokine production within CD4 T cells treated in Th1 polarizing conditions in vitro. Utilizing various combinations of the cytokines IL-12 and IL-27. They generate and present original RNA and ATAC-seq datasets to elucidate the co-dominant role of *Prdm-1 *(Blimp-1) and *Maf *(c-Maf) in regulating expression of IL-10 in Th1 cells, and this is reinforced and affirmed using mice with T-cell specific deletion of these transcription factors. This is similar (in some ways) to previous studies that used public and in-house transcriptional datasets to identify Prdm-1 and Maf as co-regulators of IL-10 production by Tr1 cells differentiated in vitro with IL-27[AO1] . Our understanding of the molecular mechanisms that underly the integration of cytokine signals within a cell and drive functional cell states is continues to develop and this study provides a piece of the puzzle through their comprehensive analysis of the integration of signals for the IL-12 family members at the transcriptional and epigenetic level. The undertaking to generate and analysis high-dimensional datasets is no easy task and the breadth of the undertaking really makes this a nice resource data set. This iteration does distinguish itself from the previous report on Prdm-1 and Maf regulating IL-10 expression in Tr1 cells and its genuinely important to know what is robust and reproducible so there is clear value to this report. The authors do try to get the most out of their novel datasets, but in places the manuscript is dense and it might help to remove redundant figures and focus the narrative to drive home their points that Prdm-1 and Maf are regulators of IL-10 expression in a variety of conditions. However, as currently written the difference in transcriptional regulation between Tr1 and Th1 cells is unclear and the data sets need to be more fully synthesized. If I was reviewing this for a colleague – my suggestion would be to look again at the original sentence at the end of the first paragraph of the Introduction. “However, the transcriptional mechanisms that regulate cytokine production by Th1 cells including their production of IL-10 are as yet unclear.” ***Thank you to the Reviewer for pointing this out. With respect to this original sentence at the end of the first paragraph of the Introduction copied above, we have expanded, rearranged and clarified what the underlying questions in the field are also quoting a relevant published review and paper*** ***as indicated below and with changes to the text of the manuscript*** ***.***

 Maybe consider re-working the sentiment that the transcriptional mechanisms that regulate cytokine production by Th1 cells including their production of IL-10 are as yet unclear. It undersells what is a rich literature on this topic that for example led to the identification of T-bet and how STATs promote Th1 responses and IL-10 production. Maybe this can be used to better frame how the current study moves the field. ***We agree that this was not clear and have now clarified the questions and current literature by adding the following paragraph in the Introduction as follows:*** ***“*** ***The transcription factors that regulate the differentiation and function of Th1 cells producing Ifng are well established, including STAT molecules and T-bet, however, whether these transcription factors regulate Il10 expression as part of the differentiation pathway of Th1 cells has been difficult to decipher (Currently Reference 9: Gabrysova et al., 2014, Current Topics Micro&Imm).*** ***Whether transcription factors such as Prdm1 or Maf, which positively induce Il10 expression, do so as part of the differentiation pathway of Th1 cells, or alternatively, simultaneously negatively regulate proinflammatory cytokines in Th1 cells, therefore not contributing to Th1 differentiation but instead reinforcing a regulated Th1 response, is unclear (Gabrysova, ref 9, 2014; Gabrysova, Ref. 16, 2018).*** ***”*** ***.***

 The comments below are provided to help with clarity and are not meant to be prescriptive and new experiments or reanalysis is not requested. ***Thank you for the useful feedback which we have attended to.***

 Figure 1 – no comments. Favorite figure. ***We are pleased that the reviewer appreciates this figure.***

 Figure 2 – These transcriptional data have already been presented within Figure 1, the only difference is the visualization used to display the data. The authors have already presented these genes and determined them to be DEGs in Figure 1C, and we are not sure how/if presenting the read counts provides any new insight. The authors already show a pearson’s correlation in Figure 1e and use the second half of Figure 2 to show correlations derived from a linear regression. It is unclear whether these graphs provide a novel lens to interpret these data. Perhaps Figure 1 is sufficient for the claims made in Figure 2? ***We thank the Reviewer for pointing out that our data were seemingly repetitive, and we have now clarified the text to describe the added value of Figure 2a and b, over-and-above what was provided in Figure 1. We have however removed Figure 2c to clarify our message. This clarification below has been added to the manuscript text:*** ***“Of transcription factors previously reported to regulate Il10 expression, only expression of Prdm1 and Maf, was significantly increased under IL-12+IL-27 and IL-27 alone conditions only, reaching maximal levels when Il10 expression was observed at days 3 and 4 and showing the strongest correlation with Il10 expression, while their expression was not observed under IL-12 or medium alone conditions (Figure 2a). Importantly expression of Batf, previously suggested to regulate Il10 in Th2 cells (Tussiwand, Nature, original reference 75), which was not revealed as correlating with Il10 expression in the analysis in Figure 1e, did not correlate with Il10 expression under IL-12+IL-27 and IL-27 alone conditions, but rather was maximally expressed on days 1 and 2, rapidly diminishing by days 3 and 4, while it’s expression increased with time in IL-12 alone differentiated Th1 cells which did not express Il10 (*** ***Figure 2b*** ***), therefore suggesting a broader function than the regulation of Il10 (*** ***Figure 2b*** ***). Other transcription factors that have been associated with Il10 gene expression including Hif1a and Nfil3*** ***^13^*** ***^,^*** ***^16^*** ***, which although expressed under IL-12+IL-27 and IL-27 alone conditions, showed less of a correlation with Il10 expression, and were found to increase with time in Th1 cells differentiated with IL-12 only which do not express Il10, therefore again suggesting a broader role for these transcription  factors in Th1 differentiation ( Figure 2b[AO2] )”.*** ***We trust this simplification of Figure 2 and this clarification of the text will satisfy the Reviewer, particularly given the nature of this manuscript submission as a Resource, and secondly since we cannot add Supplementary figures or data not shown.***

 Figure 3 – the authors take advantage of a software package BaGFoot to analysis ATAC-seq data but there is not sufficient description of the package to allow a reader to interpret the graphs. The authors highlight that these depictions show increased transcriptional activity for Prdm1 and Maf, but does this provide additional insight beyond what is shown by figure 1. Perhaps just needs to be better integrated. ***We apologise for the lack of clarity of the BaGFoot analysis of the ATAC-seq data and have expanded the description of the package accordingly as well as clarifying the text regarding the results. We again believe that these data add support at the level of transcriptional activity, over-and-above that which is provided in Figure 1. Moreover, since the manuscript was submitted as a Resource, and these data cannot be provided as a Supplementary Figure, we believe they are important to show and described at the level we have now done, in order not to detract but rather support the main story of the manuscript. These data will then be accessible for further analyses to specialist readers with further interest as the ATAC-seq data will be deposited in the GEO for public access. We have replaced the text as shown below to clarify as the Reviewer suggested.*** ***“To further investigate global changes in transcriptional activity in CD4 ^+^ naïve T cells cultured as above, we used the assay for transposase-accessible chromatin plus sequencing (ATAC-seq) to reveal functionally active genomic regions at days 2 and 3, timepoints, which marked key transcriptional changes during the differentiation of CD4 ^+^ naïve T cells into Th1 cells ( cultured as in Figure 1a). The ‘bivariate genomic footprinting’ (BaGFoot) software ^39^ was applied to the ATAC-seq data, to detect global changes in transcription factor binding activity (genome-wide) occurring between day 2 and day 3 under the different conditions. Differences in binding activity are assessed by BaGFoot software by quantifying the differences in Tn5 transposition within a transcription factor motif, by measuring the ‘footprint depth’ (Figure 3, y axis) and ‘flanking accessibility’ (Figure 3, x axis) and comparing these metrics between timepoints. A transcription factor bound to chromatin has a high footprint depth and a high flanking accessibility.*** ***Only IL-27 and IL-12+IL-27 cultured T cells showed increased transcriptional activity for Prdm1 (higher activity at day 2 vs day 3) and Maf (higher activity at day 3 vs day 2), reinforcing a role for these transcription factors in regulating transcriptional programs in IL-12+IL-27 and IL-27 cultured T cells. On the other hand, the AP-1 family members, e.g. Jun and Fos, showed increased transcriptional activity at day 3 across all cytokine differentiation conditions including medium control (Figure 3) suggesting a broader role in T helper cell activation/differentiation. Batf transcriptional activity was only evident in cells cultured in medium alone or IL-12; and Stat 3, 4 and 5 transcriptional activity was only detected under IL-12 conditions, which were not accompanied by Il10 gene expression, again implicating these transcription factors in broader roles in Th1 cell activation/differentiation. Detection of STAT activity and a transcriptome more similar to T cells cultured in medium or in IL-12 alone at days 1 and 2 (Figure 1a), suggests that Th1 cells cultured with IL-12, which do not express Il10 were temporally and qualitatively different with respect to global transcriptional activity, to Th1 cells cultured with IL-12+IL-27 and IL-27 alone, where both conditions lead to Il10 expression, although proinflammatory cytokine expression was only observed in IL-12+IL-27 driven Th1 cells.”***

 Figure 4 – Good as is. We like the figure and the written description. ***Thank you.***

 Figure 5 – The authors move onto T-cell specific depletion of Prdm-1 and Maf and transcriptional analysis of these cells via RNA-seq. Is the variance (G-I) redundant with the graphs (D-F)? ***We have modified Figure 5 and removed Figure 5g-i as suggested by the reviewer and corrected Figure 5 in the manuscript text accordingly. We thank the Reviewer for pointing this out as it clarifies the figure by removing redundancy.***

 Figure 6 – all of these genes are already shown in Figure 5 and seems redundant. ***We respectfully believe that these data are important to keep in the manuscript since they provide supporting data with respect to pathways involved which were discussed at various sections of the text. We argue that this is important to include, in view of the fact that Wellcome Open Research does not allow Supplementary Figures, or data not shown, and will be of value to researchers studying this specific area of transcriptional regulation of cytokines, especially since the manuscript is submitted as a Resource.***

 Figure 7 – The interpretation of the PCA analysis for this ATAC-seq data is similar to what has been previously established, although it is important to see the consistency. The authors go on to say that there is no chromatin remodeling, but there are differences in the visualizations of the heatmap. For example. it appears that the absence of Maf alone induces a similar chromatin remodeling to the absence of both Prdm1 and Maf. Are we interpreting this wrong? ***We are assuming that the Reviewer*** ***’*** ***s comment*** ***“*** *is similar to what has been previously established* ***”*** ***refers to Figure 1 PCA which was RNA-seq of wild type mouse T cells. We emphasise that the PCA of ATAC-seq data in Figure 7 is distinct from the PCA plot of the RNA-seq data in Figure 1 which shows cytokine conditions only, but no knockouts, whereas this Figure 7 is ATAC-seq data PCA of T cells at kinetic points of cytokine-driven conditions. The only other figure in the manuscript showing ATAC-seq data is Figure 3, which again has only cytokine conditions and no knockouts. Figures 5 and 6 are RNA-seq data showing Spearman, Singular Value Decomposition*** ***(SVD)*** ***and then clustering of only differentially expressed genes by RNA-seq*** ***not showing*** ***a PCA plot. The ATAC-seq PCA data and Spearman in Figure 7 is of ATAC-seq data, and shows T cells driven under different cytokine conditions from the different T cell-specific transcription deleted mice*** ***–*** ***Figure 7 a, b and c, PCA, and then, Figure 7b,c and d, Spearman. We firstly shortened the sentence to clarify our message and secondly added an extra line in bold below to our text referring to data in Figure 7 to clarify these findings. A major point of our findings*** ***is:*** ***“I*** ***t would appear*** ***from our findings*** ***that cytokines are the main drivers of changes in chromatin remodelling while changes in chromatin remodelling are not observed in the absence of Prdm1, Maf or both transcription factors,*** ***as we have previously*** ***reported*** ***in a different context*** ***for c-Maf*** ***^20^*** ***”*** ***.*** ***We have now added these lines to the text of our manuscript to summarise and clarify the take-home message of our findings. W*** ***e trust*** ***that this*** ***will now clarify these issues*** ***.*** **“**Moreover, major chromatin remodelling occurring between Day 2 and Day 3 was observed in all *in vitro* differentiated CD4 ^+^ T cells in the presence of cytokines. **However, no major **changes in accessibility were observed upon *Cd4* ^Cre^-mediated deletion of *Prdm1*, *Maf,* or both *Prdm1* and *Maf* ( Figure 7a–f). Together these results highlight a role for IL-27 (including IL-27 plus IL-12) in influencing the open chromatin landscape of *in vitro* differentiated CD4 ^+^ T cells and suggests that c-Maf and Blimp-1 do not drive chromatin remodelling in order to perform their gene regulation functions, as has been shown for c-Maf ^20^ . ***It*** ***would appear from our findings that cytokines are the main drivers of changes in chromatin remodelling while changes in chromatin remodelling are not observed in the absence of Prdm1, Maf or both transcription factors (Figure 7a-f). This indicates that Blimp-1 and c-Maf do not themselves induce chromatin remodelling in these differentiating Th1 cells, as previously reported for c-Maf in Th17 cells in vitro ^20 ^and as we have reported for c-Maf in ex-vivo T cells from infection models in other contexts*** ***^16^*** ***”*** ***.***

 Figure 8 – Maybe there is a mis-understanding (on our part). How does the IL-10 and IFN-g locus look in the ATAC-seq data generated from T -cell specific depletion of Prdm-1 and Maf as shown in Figure 7. These data would be more interesting to see rather than the WT cells (described in the figure legend?). ***The data on the IL-10 and IFN-g loci are from the WT T cells differentiated under the different cytokine conditions only, as those in T cells bearing T -cell specific depletion of Prdm-1 and Maf, or both, showed no difference in terms of chromatin accessibility as compared to controls*** ***–*** ***this is why we did not show them. We have added the line below to the text of the manuscript to clarify this point.*** ***“*** ***These accessible binding sites were not affected by T -cell specific depletion of Prdm-1 and Maf, or both transcription factors, as compared to controls.*** ***”***

 Fig 9, 10 and 11. The authors have done a lot of analysis but it would be genuinely helpful if they could provide clarity in the discussion as to why these genes are interesting. For example, the focus on Cluster 5 is unclear. ***Figure 9 provides the schematic of the integration of the multiomic data.*** ***The reason that specific clusters were chosen, (from Figure 5g and 5j) for depiction as gene regulatory networks in Figures 10 and 11, is because these clusters contained direct targets of Blimp-1 and/or c-Maf and were the most affected at the RNA expression level upon Cd4Cre-mediated deletion of Prdm1, Maf, or both Prdm1 and Maf eg. for IL-12+IL-27 found in Clusters 2,1,7 in Figure 5g; and for IL-27 alone found in Clusters 5, 7, 1 in Figure 5j, We now provide an explanation at the beginning of the data of why these clusters were selected:*** ***“*** ***S*** ***pecific clusters from the RNA expression data (Figure 5g and*** ***5*** ***j) were chosen for our*** ***depiction as gene regulatory networks in Figures 10 and 11,*** ***because*** ***these clusters contained*** ***direct targets of Blimp-1 and/or c-Maf*** ***that*** ***were the most affected at the RNA expression level upon Cd4Cre-mediated deletion of Prdm1, Maf, or both Prdm1 and Maf eg. for IL-12+IL-27 found in Clusters 2,1,7 in Figure 5g*** ***;*** ***and for IL-27 alone found in Clusters 5, 7, 1 in Figure 5j.*** ***”***

**We thank the reviewer for the helpful comments for clarifying our manuscript.**

# Reviewer report: Reviewer response for version 1

**DOI:** 10.21956/wellcomeopenres.21798.r67171

**Published:** 2023-10-05

**Authors:** Leonie Taams

**Affiliations:** 1 King's College London, London, England, UK

This manuscript compellingly demonstrates that Maf and Blimp1 are transcriptional regulators of both Il10 and Ifng gene expression during mouse naïve CD4+ T cell differentiation in the presence of IL-12 and IL-27. Whilst both Maf and Blimp1 had been associated previously with positive regulation of Il10 expression, the results presented here demonstrate that they in parallel negatively regulate Ifng, as well as other Th1-related effector molecules including Il2 and Id2.

 The data presented in the paper consist of RNAseq, ATACseq and qPCR data of naïve CD4+ T cells differentiated in the absence or presence of IL-12, IL-27 or IL-12 plus IL-27, using cells from either wt or from CD4+ T cell-conditional KO mice for Maf, Prdm1 or the combination.

 The authors employ an impressive range of sophisticated and complex computational modelling tools to analyse the datasets. This reviewer is not qualified to comment on these tools or the analysis approach; input from a qualified bioinformatician expert would be valuable in this respect. I will therefore focus my comments on the overall concept and interpretation of the results of the study.

**Abstract:**

 It would be useful to indicate clearly in the abstract that the data relate to mouse CD4+ T cells only.

**Introduction:**

 It would help to more clearly spell out the differences in experimental design of the starting material (i.e. the cell culture conditions) between the approach taken in this manuscript and ref 13, as readers who are less familiar may not immediately appreciate how the two studies differentiate.

 Results page 9, relating to figure 1d: The authors comments *“[…] expression of Il10 in IL-12+IL-27 and IL-27-driven CD4+ T cells appeared to correlate strongly with expression of Prdm1 as well as Maf over time, while Ifng expression appeared to be repressed at peak times under these conditions…”.* I think that based on the data in Figure 1d, ‘repressed’ is too strong a word to use; ‘reduced’ seems to be more fitting.

 Figure 4: the authors state “ *Th1 cells differentiated with IL-12+IL-27 and IL-27-driven T cells showed significant levels of Il10 expression, which was diminished in the absence of Prdm1 or Maf, and to the greatest extent in the absence of both transcription factors (Figure 4a–c).” *I would suggest to remove the final part “and to the greatest extent in the absence of both transcription factors” as this is not that obvious from the actual figure: when comparing Fig 4a and 4b the data for IL10 are virtually similar for the double KO and the Prdm1 single KO cells.

 Figures 5 & 6: these figures are the result from highly complex bioinformatical analysis approaches and I found both the narrative and the depiction difficult to understand and interpret. Whilst the complexity and sophistication of the approach are to be applauded and with the appreciation that for some readers these figures will contain valuable information and data, this reviewer wonders to what extent these sections help versus hinder the clarity of the manuscript. In other words, are the analysis and its associated narrative and depictions essential to go from Figure 4 to Figure 7? Could the information perhaps be shortened and placed as Suppl data instead?

 Figure 11: the added value is not entirely clear to this reviewer, and given that the narrative on this figure is rather short, I wonder if this could be removed. 

**Discussion:**

 It would be very informative if the authors could add a specific section in the discussion highlighting to what extent c-Maf and Blimp-1 are known to regulate *IL10 *and *IFNG* expression in human CD4+ T cells, under similar conditions or otherwise, and/or what the gaps in knowledge are in this context with respect to human CD4+ T cells.

 There are occasions where the authors a bit too strongly exclude a role for certain transcription factors that previously were found to regulate IL10 expression. An example can be found here (discussion page 22):  “ *Likewise, the transcription factor Batf, which has been previously reported to regulate Il10 expression in differentiating Th2 cells was actually downregulated in both IL-12 plus IL-27 and IL-27 alone differentiation conditions, whilst increasing under IL-12 alone differentiation conditions, and showed very poor correlation with Il10 expression. Thus, as discussed earlier for IL-21, it is likely that a number of these transcription factors are required for the differentiation and possibly proliferation of T helper cells rather than for direct positive regulation of Il10 gene expression.”*

 It is also possible that these other transcriptional regulators have the capacity to regulate Il10, but that this is cell and/or context dependent, i.e. depending on the naïve/effector state of the cell, their polarisation or differentiation. In other words, it would be helpful to acknowledge that the regulation of Il10 and Ifng by Blimp-1 and c-Maf as described in the manuscript is tested specifically and solely in the context of the two cytokines and their combination in mouse naïve CD4+ T cells.

 Finally, a short section on the limitations of this study would be helpful, i.e. acknowledge that this work is all done at the transcriptional level, and that it has not been formally tested that Blimp-1/c-Maf overexpression alone or in combination leads to direct upregulation of IL-10 and downregulation of IFNg at the protein level.

**Minor:**

 Page 3: the following sentence is very long and not very clear: “Although IL-27 regulates the effector function of IL-12 driven Th1 cells, producing high levels of IFN-γ, by inducing the production of IL-10 to limit and control Th1-mediated immunopathology in vivo12,14,15,27, the mechanisms of transcriptional regulation of Il10 and proinflammatory cytokines in Th1 cells are unclear.”

Is the work clearly and accurately presented and does it cite the current literature?

Partly

If applicable, is the statistical analysis and its interpretation appropriate?

I cannot comment. A qualified statistician is required.

Are all the source data underlying the results available to ensure full reproducibility?

Yes

Is the study design appropriate and is the work technically sound?

Yes

Are the conclusions drawn adequately supported by the results?

Yes

Are sufficient details of methods and analysis provided to allow replication by others?

Yes

Reviewer Expertise:

Immunoregulation (wet-lab based) with limited expertise in bioinformatic analysis

I confirm that I have read this submission and believe that I have an appropriate level of expertise to confirm that it is of an acceptable scientific standard, however I have significant reservations, as outlined above.

# Response

**Published:** 2023-11-23

**Authors:** Anne O'Garra

**Affiliations:** The Francis Crick Institute, UK

**Leonie Taams ** King's College London, London, England, UK

 This manuscript compellingly demonstrates that Maf and Blimp1 are transcriptional regulators of both Il10 and Ifng gene expression during mouse naïve CD4+ T cell differentiation in the presence of IL-12 and IL-27. Whilst both Maf and Blimp1 had been associated previously with positive regulation of Il10 expression, the results presented here demonstrate that they in parallel negatively regulate Ifng, as well as other Th1-related effector molecules including Il2 and Id2. The data presented in the paper consist of RNAseq, ATACseq and qPCR data of naïve CD4+ T cells differentiated in the absence or presence of IL-12, IL-27 or IL-12 plus IL-27, using cells from either wt or from CD4+ T cell-conditional KO mice for Maf, Prdm1 or the combination. The authors employ an impressive range of sophisticated and complex computational modelling tools to analyse the datasets. This reviewer is not qualified to comment on these tools or the analysis approach; input from a qualified bioinformatician expert would be valuable in this respect. I will therefore focus my comments on the overall concept and interpretation of the results of the study.

**Abstract: ** It would be useful to indicate clearly in the abstract that the data relate to mouse CD4+ T cells only. **Thank you this has been corrected.**

**Introduction: ** It would help to more clearly spell out the differences in experimental design of the starting material (i.e. the cell culture conditions) between the approach taken in this manuscript and ref 13, as readers who are less familiar may not immediately appreciate how the two studies differentiate. ***Thank you to the Reviewer, this is a good point and we have introduced text at appropriate stages of the Introduction and Results to indicate what is different and thus what are the advances of our study over the published Ref 13 study.*** ***Firstly, we have rearranged the Introduction, to spell out the question at the end of the first paragraph regarding the regulation of Il10 expression in Th1 cells and re-writing the lines to spell out our question more clearly; this text had been originally included at the end of the paragraph describing the findings of Ref. 13, but is better placed here at the beginning of our Introduction:*** ***“*** ***The transcription factors that regulate the differentiation and function of Th1 cells producing Ifng are well established, including STAT molecules and T-bet, however, whether these transcription factors regulate Il10 expression as part of the differentiation pathway of Th1 cells has been difficult to decipher (Gabrysova et al., 2014, Current Topics Micro&Imm; Currently Reference 9:). Whether transcription factors such as Prdm1 or Maf, which positively induce Il10 expression, do so as part of the differentiation pathway of Th1 cells, or alternatively, simultaneously negatively regulate proinflammatory cytokines in Th1 cells, therefore not contributing to Th1 differentiation but instead reinforcing a regulated Th1 response, is unclear (Gabrysova, ref 9, 2014; Gabrysova, Ref. 16, 2018)”.*** **Then at the end of the 3 ^rd^ paragraph describing the Ref 13 study we have now added this line:** **“** **The role of *Prdm1* and *Maf* in regulating *Il10 *and proinflammatory cytokines in IL-10-producing Th1 cells has as yet not been reported.”** **And in the last paragraph of the Introduction, we have added lines to the text (shown in *bold italics below*), within the original final paragraph of the Introduction, to clarify our approach and therefore our novel findings: **“In this study we applied computational analysis of gene regulation derived from temporal profiling of gene expression clusters integrated with analysis of active genomic regions in CD4 ^+^ Th1 effector cells differentiated with IL-27 ***plus IL-12, which express Il10 together with proinflammatory cytokines. Gene expression and active genomic region analysis data was compared to T cells differentiated in IL-27 alone (named “Tr1 cells”*** ***^13^*** ***)*** ***, which express Il10 but no proinflammatory cytokines, or conversely IL-12 alone, which express proinflammatory cytokines but not Il10, all compared to medium control, which do not express cytokines. The aim was to elucidate the transcriptional network of genes controlling expression of Il10 and pro-inflammatory effector genes in Th1 cells, and identify transcription factors that not only induced Il10, and additionally negatively regulated Th1 proinflammatory gene expression,*** ***and were therefore not part of the Th1 differentiation pathway*** **. **We show that the transcription factors Blimp-1 and c-Maf each have unique and common effects on cytokine gene regulation and not only co-operate to induce *Il10* gene expression in IL-12 plus IL-27 differentiated Th1 cells, but additionally directly negatively regulate key proinflammatory cytokines including *Ifng*, thus providing mechanisms for reinforcing the regulation of Th1 cell responses. Thus, Blimp-1 and c-Maf positively and negatively regulate a network of both unique and common anti-inflammatory and pro-inflammatory genes to reinforce a Th1 response that will allow eradication of pathogens with minimum immunopathology. “ ***We have also now clarified our culture conditions at the beginning of our Results section, which together with our rationale now provided at the end of the Introduction we believe now clarifies our approach and its difference with that of the Ref 13 study – leading then to novel findings:*** ***“To achieve this, we first analysed changes in temporal gene expression in vitro in naïve CD4 ^+^ T cells stimulated with anti-CD3 and anti-CD28 as described in the Methods, and differentiated these over time with IL-27 plus IL-12 into CD4 ^+^ Th1 effector cells which express Il10 together with proinflammatory cytokines such as Ifng, and compared to T cells differentiated in IL-27 alone (named “Tr1 cells”*** ***^13^*** ***)*** ***, which express Il10 but no proinflammatory cytokines, or conversely IL-12 alone, which express proinflammatory cytokines including Ifng but not Il10, all compared to medium control, which do not express cytokines.”***

 Results page 9, relating to figure 1d: The authors comments *“[...] expression of Il10 in IL-12+IL-27 and IL-27-driven CD4+ T cells appeared to correlate strongly with expression of Prdm1 as well as Maf over time, while Ifng expression appeared to be repressed at peak times under these conditions...”. *I think that based on the data in Figure 1d, ‘repressed’ is too strong a word to use; ‘reduced’ seems to be more fitting. ***Thank you – we have changed the word “repressed” to “reduced”.***

 Figure 4: the authors state “ *Th1 cells differentiated with IL-12+IL-27 and IL-27-driven T cells showed significant levels of Il10 expression, which was diminished in the absence of Prdm1 or Maf, and to the greatest extent in the absence of both transcription factors (Figure 4a–c).” *I would suggest to remove the final part “and to the greatest extent in the absence of both transcription factors” as this is not that obvious from the actual figure: when comparing Fig 4a and 4b the data for IL10 are virtually similar for the double KO and the Prdm1 single KO cells. ***We thank the Reviewer, we have removed “and to the greatest extent in the absence of both transcription factors” from the “final part “of this sentence***.

 Figures 5 & 6: these figures are the result from highly complex bioinformatical analysis approaches and I found both the narrative and the depiction difficult to understand and interpret. Whilst the complexity and sophistication of the approach are to be applauded and with the appreciation that for some readers these figures will contain valuable information and data, this reviewer wonders to what extent these sections help versus hinder the clarity of the manuscript. In other words, are the analysis and its associated narrative and depictions essential to go from Figure 4 to Figure 7? Could the information perhaps be shortened and placed as Suppl data instead? ***Unfortunately, Wellcome Open Research does not allow for Supplementary Figures and hence the inclusion here in Main figures. We believe that these data are important to keep in the manuscript since they provide supporting data with respect to firstly the genes reduced in the different transcription factor deleted T cells (Figure 5). Also the clusters in Figure 5 are of fundamental importance to Figures 10 and 11. In fact we have added extra text on the rationale for why specific clusters were chosen, (from Figure 5g and 5j) for depiction as gene regulatory networks in Figures 10 and 11, which is because these clusters contained direct targets of Blimp-1 and/or c-Maf and were the most affected at the RNA expression level upon Cd4Cre-mediated deletion of Prdm1, Maf, or both Prdm1 and Maf eg. for IL-12+IL-27 found in Clusters 2,1,7 in Figure 5g; and for IL-27 alone found in Clusters 5, 7, 1 in Figure 5j, We now provide an explanation at the beginning of the data regarding Figures 10 and 11 as to why these clusters were selected which shows the importance of keeping Figure 5, although we have however simplified Figure 5 by removing Fig 5g-i.*** ***“*** ***S*** ***pecific clusters from the RNA expression data (Figure 5g and*** ***5*** ***j) were chosen for our*** ***depiction as gene regulatory networks in Figures 10 and 11,*** ***because*** ***these clusters contained*** ***direct targets of Blimp-1 and/or c-Maf*** ***that*** ***were the most affected at the RNA expression level upon Cd4Cre-mediated deletion of Prdm1, Maf, or both Prdm1 and Maf eg. for IL-12+IL-27 found in Clusters 2,1,7 in Figure 5g*** ***;*** ***and for IL-27 alone found in Clusters 5, 7, 1 in Figure 5j.*** ***”*** ***Secondly, we believe the pathways involved (Figure 6), which were discussed at various sections of the text, should be kept especially since Wellcome Open Research does not allow Supplementary Figures, or data not shown, and we believe these Figures will be of value to researchers studying this specific area of transcriptional regulation of cytokines, especially since the manuscript is submitted as a Resource, and will point them to data which they could further analyse which will be deposited in the GEO for public use.***

 Figure 11: the added value is not entirely clear to this reviewer, and given that the narrative on this figure is rather short, I wonder if this could be removed. **The reason for including Figure 11 is to compare with Ref 13, Zhang et al., 2020, which we did describe and discuss in the text – since those in Figure 10 were distinct from those described in Ref. 13, again this was discussed.  Again we believe it is important to keep this figure and text since *Wellcome Open Research does not allow Supplementary Figures, or data not shown, and especially since the manuscript is submitted as a Resource, which could be further analysed as all will be deposited in the GEO for public use, but showing the figures is essential to guide the reader to the GEO data, as well as for the content in itself.***

**Discussion: ** It would be very informative if the authors could add a specific section in the discussion highlighting to what extent c-Maf and Blimp-1 are known to regulate *IL10 *and *IFNG *expression in human CD4+ T cells, under similar conditions or otherwise, and/or what the gaps in knowledge are in this context with respect to human CD4+ T cells. **We have added the paragraph below to the Discussion, with the listed references – to be included - as marked in the text - by Editorial office.** ***Relevant to our study, in recent years Blimp-1 and c-Maf have been associated with transcriptional signatures from human disease such as colitis and rheumatoid arthritis (Mijnheer, 2021; Ellinghaus, 2013). The conserved nature of these transcription factors between mouse and humans suggests similar transcriptional mechanisms for cytokine gene regulation operate in mouse and humans. In support of this, SNPs in Blimp-1 have been associated with elevated IFN-g expression in colitis patients (Ellinghaus, 2013) and Blimp-1 has been shown to bind conserved CNS sites in human and mouse in the Ifng/IFNG and Tbx21/TBX21 loci in T cells and NK cells (Cimmino, 2008; Smith, 2010). Likewise, c-Maf has been shown to be regulated by the cholesterol pathway and regulate IL10 expression by human Th1 cells (Perucha, 2019) in keeping with regulation of Il10 expression in mouse Th1 cells as we report herein. Very recently both Blimp-1 and c-Maf have been reported to co-regulate CD4 ^+^ T cell derived IL-10 in Crohn*** ***’*** ***s patients (Ahlers, 2022) and identified in Th1/Tr1 cells from malaria patients (Nifeffer, 2023; Edwards, 2023). However, the exact dynamic both Blimp-1 and c-Maf play in the regulation of the expression of cytokines, and other important inflammatory genes needs further research, particularly given that there is a lack of ChIP-seq datasets from individual Th-cell subsets for both of these transcription factors in both mouse and human.***  

- Mijnheer, G. *et al.* Conserved human effector Treg cell transcriptomic and epigenetic signature in arthritic joint inflammation. *Nat Commun* **12**, 2710, doi:10.1038/s41467-021-22975-7 (2021).

- Ellinghaus, D. *et al.* Association between variants of PRDM1 and NDP52 and Crohn's disease, based on exome sequencing and functional studies. *Gastroenterology* **145**, 339-347, doi:10.1053/j.gastro.2013.04.040 (2013).

- Cimmino, L. *et al.* Blimp-1 Attenuates Th1 Differentiation by Repression of ifng, tbx21, and bcl6 gene expression. *The Journal of Immunology* **181**, 2338-2347, doi:10.4049/jimmunol.181.4.2338 (2008). **Was referenced in first submission, needs re-referencing.**
- 21  Cimmino L, Martins GA, Liao J, et al.: Blimp-1 Attenuates Th1 Differentiation by Repression of *ifng, tbx21*, and *bcl6* gene expression. *J Immunol.* 2008;181(4):2338–2347. 18684923 10.4049/jimmunol.181.4.2338  

- Smith, M. A. *et al.* PRDM1/Blimp-1 controls effector cytokine production in human NK cells. *J Immunol* **185**, 6058-6067, doi:10.4049/jimmunol.1001682 (2010).
- Perucha, E. *et al.* The cholesterol biosynthesis pathway regulates IL-10 expression in human Th1 cells. *Nat Commun* **10**, 498, doi:10.1038/s41467-019-08332-9 (2019). **Was referenced in first submission – needs re-referencing.**
- 74  Perucha E, Melchiotti R, Bibby JA, et al.: The cholesterol biosynthesis pathway regulates IL-10 expression in human Th1 cells. *Nat Commun.* 2019;10(1): 498. 30700717 10.1038/s41467-019-08332-9 6353904

- Ahlers, J. *et al.* A Notch/STAT3-driven Blimp-1/c-Maf-dependent molecular switch induces IL-10 expression in human CD4(+) T cells and is defective in Crohn s disease patients. *Mucosal Immunol* **15**, 480-490, doi:10.1038/s41385-022-00487-x (2022).

- Edwards, C. L. *et al.* IL-10-producing Th1 cells possess a distinct molecular signature in malaria. *J Clin Invest* **133**, doi:10.1172/JCI153733 (2023).

- Nideffer, J. & Jagannathan, P. Type I regulatory T cells in malaria: of mice and men. *J Clin Invest* **133**, doi:10.1172/JCI166019 (2023).

There are occasions where the authors a bit too strongly exclude a role for certain transcription factors that previously were found to regulate IL10 expression. An example can be found here (discussion page 22): “ *Likewise, the transcription factor Batf, which has been previously reported to regulate Il10 expression in differentiating Th2 cells was actually downregulated in both IL-12 plus IL-27 and IL-27 alone differentiation conditions, whilst increasing under IL-12 alone differentiation conditions, and showed very poor correlation with Il10 expression. Thus, as discussed earlier for IL-21, it is likely that a number of these transcription factors are required for the differentiation and possibly proliferation of T helper cells rather than for direct positive regulation of Il10 gene expression.” * It is also possible that these other transcriptional regulators have the capacity to regulate Il10, but that this is cell and/or context dependent, i.e. depending on the naïve/effector state of the cell, their polarisation or differentiation. In other words, it would be helpful to acknowledge that the regulation of Il10 and Ifng by Blimp-1 and c-Maf as described in the manuscript is tested specifically and solely in the context of the two cytokines and their combination in mouse naïve CD4+ T cells. ***We thank the reviewer for pointing this out. We have amended the text in the Discussion accordingly:*** ***“*** ***Alternatively, the capacity of these*** ***transcription factors to regulate Il10 may be cell and/or context dependent, for example regulating Il10 in some effector T cells and not others, as previously discussed*** ***(Gabrysova, ref 9, 2014; Gabrysova, Ref. 16, 2018).*** ***[AO1]*** ***”***

 Finally, a short section on the limitations of this study would be helpful, i.e. acknowledge that this work is all done at the transcriptional level, and that it has not been formally tested that Blimp-1/c- Maf overexpression alone or in combination leads to direct upregulation of IL-10 and downregulation of IFNg at the protein level. ***We thank the Reviewer for this suggestion.  We have added the following text to our Discussion to address this issue.*** ***“A limitation of this study is firstly that the regulation of Il10 versus proinflammatory cytokines by Blimp-1 and c-Maf was firstly only demonstrated at a transcriptional level and consequent effects on protein production were not the focus of the study. Secondly, the effects of T cell-specific deletion of Prdm1 and Maf on Il10 and proinflammatory cytokine expression was only investigated in vitro. Our continuing studies will further address the role of Blimp-1 and c-Maf in regulation of cytokine responses in vivo, and the physiological consequences of T cell-specific deletion of these transcription factors in response to pathogens and/or pathobionts”.***

**Minor: ** Page 3: the following sentence is very long and not very clear: “Although IL-27 regulates the effector function of IL-12 driven Th1 cells, producing high levels of IFN-γ, by inducing the production of IL-10 to limit and control Th1-mediated immunopathology in vivo12,14,15,27, the mechanisms of transcriptional regulation of Il10 and proinflammatory cytokines in Th1 cells are unclear.” ***We thank the Reviewer for pointing this out. We have shortened this sentence into two sentences as suggested but also added more text to answer issues raised by the other Reviewer regarding the latter half of this sentence and the question being addressed in this study.*** **Is the work clearly and accurately presented and does it cite the current literature? ** Partly ***We have added more text to clarify the questions being addressed in this study in the Introdcution.*** ***We have added a paragraph to the Discussion, with reference to the literature in human, with the listed references – to be added by Editorial office. The added text to the Discussion to address this issue is as follows*** ***“Relevant to our study, in recent years Blimp-1 and c-Maf have been associated with transcriptional signatures from human disease such as colitis and rheumatoid arthritis (Mijnheer, 2021; Ellinghaus, 2013). The conserved nature of these transcription factors between mouse and humans suggests similar transcriptional mechanisms for cytokine gene regulation operate in mouse and humans. In support of this, SNPs in Blimp-1 have been associated with elevated IFN-g expression in colitis patients (Ellinghaus, 2013) and Blimp-1 has been shown to bind conserved CNS sites in human and mouse in the Ifng/IFNG and Tbx21/TBX21 loci in T cells and NK cells (Cimmino, 2008; Smith, 2010). Likewise, c-Maf has been shown to be regulated by the cholesterol pathway and regulate IL10 expression by human Th1 cells (Perucha, 2019) in keeping with regulation of Il10 expression in mouse Th1 cells as we report herein. Very recently both Blimp-1 and c-Maf have been reported to co-regulate CD4 ^+^ T cell derived IL-10 in Crohn’s patients (Ahlers, 2022) and identified in Th1/Tr1 cells from malaria patients (Nifeffer, 2023; Edwards, 2023). However, the exact dynamic role of both Blimp-1 and c-Maf play in the regulation of the expression of cytokines, and other important inflammatory genes needs further research.”***
